# The ETFL formulation allows multi-omics integration in thermodynamics-compliant metabolism and expression models

**DOI:** 10.1038/s41467-019-13818-7

**Published:** 2020-01-13

**Authors:** Pierre Salvy, Vassily Hatzimanikatis

**Affiliations:** Laboratory of Computational Systems Biotechnology, École Polytechnique Fédérale de Lausanne (EPFL), Lausanne, Switzerland

**Keywords:** Metabolic engineering, Computational models, Data integration, Software, Metabolic engineering

## Abstract

Systems biology has long been interested in models capturing both metabolism and expression in a cell. We propose here an implementation of the metabolism and expression model formalism (ME-models), which we call ETFL, for Expression and Thermodynamics Flux models. ETFL is a hierarchical model formulation, from metabolism to RNA synthesis, that allows simulating thermodynamics-compliant intracellular fluxes as well as enzyme and mRNA concentration levels. ETFL formulates a mixed-integer linear problem (MILP) that enables both relative and absolute metabolite, protein, and mRNA concentration integration. ETFL is compatible with standard MILP solvers and does not require a non-linear solver, unlike the previous state of the art. It also accounts for growth-dependent parameters, such as relative protein or mRNA content. We present ETFL along with its validation using results obtained from a well-characterized *E. coli* model. We show that ETFL is able to reproduce proteome-limited growth. We also subject it to several analyses, including the prediction of feasible mRNA and enzyme concentrations and gene essentiality.

## Abstract (web summary)

Accounting for the effects of genetic expression in genome-scale metabolic models is challenging. Here, the authors introduce a model formulation that efficiently simulates thermodynamic-compliant fluxes, enzyme and mRNA concentration levels, allowing omics integration and broad analysis of in silico cellular physiology.

## Introduction

Metabolic modeling, which helps making sense of the metabolism in a biological network, is an important tool for engineering biocatalysts, with applications in biofuels, drug design, microbial community analysis, and personalized medicine. Model accuracy is instrumental to the success of these applications through an efficient engineering of the host organisms. However, incorporating expression information into metabolic networks poses a significant challenge, and most current models do not even attempt it—effectively excluding an important network in biological systems that can drastically affect results. In metabolic engineering, strains are modified and controlled at the genome level through the transcriptome, and the effects are observed at the fluxome level, which accounts for the range of metabolic reactions in an organism. In between these two levels is the proteome that performs the biochemical transformations according to the genetic template, though it is this middle step in the process that cannot yet be robustly and efficiently incorporated into models of metabolic systems. Because of the complex interplay between these different layers of control, understanding expression and incorporating this into future models is key for improving metabolic engineering.

Classically, model-based strain design has relied on tools that use the DNA sequence of an organism and homology with well-studied organisms to infer a network of metabolic reactions that happen inside a cell of that organism, which is called a genome-scale model (GEM). With current technologies and tools like metagenome sequencing^1^, it is possible to generate GEMs for hundreds of different species at a time. GEMs are particularly amenable to flux balance analysis (FBA), which models metabolism at the fluxome level using linear optimization techniques. However, plain FBA has been known to predict biochemically unrealistic solutions like free high-flux cycles or thermodynamically infeasible pathways. It also scales growth linearly with carbon uptake, which is not observed at high-uptake fluxes. FBA also fails to capture growth-dependent and protein-level effects, such as enzyme saturation or proteome-related limitations. Hence, several efforts have been made to supplement FBA with additional constraints to improve its predictive power. For example, thermodynamics-based flux analysis (TFA)^2,3^ uses thermodynamic constraints to enforce thermodynamically consistent reaction directionalities and to allow the integration of metabolomics. Resource balance models add a total proteome capacity constraint, as formulated in Beg et al.^4^, to model the proteome-related limitations of the cell, as enzymes have to compete for the constrained total amount of cellular proteins. Frameworks like GECKO^5^ further build on this resource balance idea and include flux constraints based on proteomics, such as $v\le {V}_{\mathrm{max}}={k}_{{{\mathrm{cat}}}}\left[E\right]$ as well as a constraint on the total proteome mass. Finally, metabolomics and expression models (ME-models)^6,7^ were the first to integrate the entirety of the expression mechanisms of the cell from the bottom-up, including mRNA and protein synthesis.

However, simultaneously accounting for all of these constraints is challenging because of the formulation of each method, as TFA models involve integer variables that yield a mixed-integer linear program (MILP), whereas ME-models involve bilinear constraints that require special optimization procedures and a high-precision (quad-precision) solver^8–10^. Mixing these methods would require the inclusion of integers in ME-models, which is not straightforward and would lead to more complex mixed-integer nonlinear programs (MINLP) that are computationally intensive to solve. In addition, the amount of RNA and protein, the RNA and protein expression rates, and their stabilities are all growth dependent^11^, and including accurate representations of these variables leads to even more complex, nonlinear models. Meanwhile, although resource balance models such as GECKO could theoretically be integrated into TFA or ME-models in the current formulations, to the best of our knowledge, no link with TFA or ME-models has been proposed. Therefore, the metabolic engineering community needs a common formulation for these methodologies to build the most accurate models.

We investigated the development of such a framework and propose herein a unified formulation for Expression and Thermodynamics-enabled FLux models (ETFL) that can account for the above integration issues. To our knowledge, ETFL is the first formulation that can account at the same time for expression, thermodynamics, and growth-dependant variables. It is also the first to do so using common double-precision MILP solvers. In ETFL, we address the compatibility of the formulations by expressing the growth rate variable in bilinear products as a piecewise constant function. We also address the issue of solver precision by performing a scaling that reduces the range of orders of magnitude of the variables. This reformulation allows us to transform the problem into a MILP, which we can solve efficiently using common open source or commercial solvers. The resulting model is then effectively able to directly integrate thermodynamic constraints as well as expression constraints and growth-dependent parameters. In this model, metabolite, enzyme, and mRNA concentration levels are explicitly defined to enable fast and easy omics integration: metabolites through their log-concentration variables in thermodynamics constraints, and enzymes and mRNA through their total concentration variables in the expression constraint. Finally, we show an application of this framework to a well-characterized *E. coli* model, iJO1366^12^.

Important assumptions are made to derive this formulation. The two most notables ones are (i) we can neglect the dilution rate of metabolites, and (ii) the steady-state approximation holds. While these assumptions are commonly made in FBA, we discuss them in details in the Supplementary Note 3, where we also assess their validity in a context where macromolecules are taken into account. Briefly, these assumptions hold because (i) the dilution rate of the metabolites is negligible in front of their synthesis and consumption rates, and (ii) the dynamics of metabolism (including expression) are faster than that of the environment of the cell.

## Results

### Formulation of the expression problem

ETFL is an ME-model implementation because it proposes a formulation that both accounts for metabolism and expression constraints. ME-models do not aim to replace kinetic models, but to account for the expression cost of making the enzymes that are necessary to carry a biochemical flux. In ETFL, this includes the cost of peptide and mRNA synthesis, as well as the competition for ribosomes and RNA polymerase in a limited proteome.

To transparently account for expression mechanisms and increase the predictive power of our models, we needed to derive the equations that could bridge the biochemistry with the optimization problem that is ETFL. Here, we present a summary of these equations, and detail their derivation in the Methods section. We derived these equations using assumptions similar to those used in the formulation of the GECKO^5^ and ME-model^6–8^.

This formulation relies on derivations rooted in the biological mechanism of expression and depends on a number of biochemical parameters related to the cell. In particular, the mass balances of the macromolecules are expressed using concentration variables. Each mass balance will yield an equation where the concentrations of the macromolecules will be variables, thus effectively formulating a new constraint of the model and allowing us to calculate concentration values by solving the model.

We can write the quasi-steady-state mass balance for macromolecules as follows:

1 $${v}_{\star }^{{\rm{syn}}}-{v}_{\star }^{{\mathrm{deg}} }-\mu * {G}_{\star }=0,$$

where $\star$ represents the indexing of the macromolecule, ${v}_{\star }^{{\rm{syn}}}$ is the synthesis term, ${v}_{\star }^{{\mathrm{deg}} }$ is the degradation term, and $\mu * {G}_{\star }$ is the dilution term. The asterisk “$*$” signifies the product of two variables. The detail of the derivation is available in the Methods section.

Using this formalism, for each macromolecule we can define and link together a synthesis flux, a degradation flux, and the macromolecule’s concentration. Knowing enzyme concentrations allows us to bound the variables representing metabolic reaction fluxes with their maximum catalytic rate according to the classical equation:

2 $$v\le {k}_{{\rm{cat}}}\cdot E,$$

where ${k}_{{\rm{cat}}}$ is the catalytic rate constant of the enzyme $E$ with respect to flux $v$. The dot product “$\cdot$” signifies here a product between a parameter value and a variable. In this same fashion, we can also constrain the synthesis flux for the peptides, which are then assembled into enzymes. Peptide synthesis is simply a metabolic reaction that consumes energy (under the form of GTP) and charged tRNAs and produces a peptide and uncharged tRNAs. The catalytic rate of the reaction is proportional to the maximum ribosomal catalytic rate divided by the length of the peptide to be synthesized. The same can be said about mRNA synthesis, which uses nucleoside triphosphates and is catalyzed by the RNA polymerase. The constraints are explained in the Methods section, in which we detail a de novo derivation of the constraint set that describe the expression problem.

The part of the matrix that has been added to the FBA problem to account for expression has been termed the expression problem (EP). Although this initial formulation is bilinear, we detail in the Methods section how we cast it to a MILP.

### Biomass reaction synthesis and mass balance

In FBA, the biomass reaction is an artificial, lumped reaction that represents the consumption of metabolites in proportion to the cell growth rate. This consumption reflects nucleoside triphosphate (NTP) requirements for mRNAs, amino acid requirements for proteins, lipid requirements for the cell wall, or metal ion needs. Biomass reaction inclusiveness depends on the modeling assumptions made during the model curation process and can vary significantly among models of the same species. The consumed amount of each metabolite is usually estimated experimentally by measuring the the amounts of these metabolites in dried cell mass. Because the stoichiometric ratios of metabolites in the biomass reaction are fixed, the abundance of metabolites is the same for all growth rates. This simplifying assumption, necessary in FBA, goes against experimental evidence. Neidhardt and Curtis^11^ report for instance that mRNA and protein mass ratios in the cell change with growth rate.

Because ETFL has explicit expression requirements through transcription, translation, and tRNA-charging reactions, it is possible to account for varying ratios of NTPs and amino acids as the growth rate changes, an effect that is captured in experiments^11^. In this context, the approximation made in FBA can be written using ETFL terms:

3 $$\forall {{\rm{aa}}}_{i},\qquad {\eta }_{{{\rm{aa}}}_{i}}^{{v}_{{\rm{biomass}}}}\cdot \mu \approx {v}_{{{\rm{aa}}}_{i}}^{{\rm{charging}}},$$

4 $$\forall {{\rm{NTP}}}_{i},\qquad {\eta }_{{{\rm{NTP}}}_{i}}^{{v}_{{\rm{biomass}}}}\cdot \mu \approx {\sum _{j\in {\mathcal{J}}}}{v}_{{{\rm{NTP}}}_{i}}^{{{\rm{tcr}}}_{j}},$$

where ${v}^{{\rm{biomass}}}$ represents the flux through the biomass equation, and ${\eta }_{{m}_{i}}^{{v}_{{\rm{biomass}}}}$ is the  stoichiometric coefficient of metabolite ${m}_{i}$ in the biomass reaction. For each metabolite participating in the biomass reaction, the expressions above are obtained by equating the corresponding mass balance constraints in ETFL and in FBA. Hence, to avoid accounting for the expression requirements twice (once through the biomass equation, once through the EP), it is necessary to remove the participation of these metabolites linked to expression from the biomass reaction.

### Summary of the formulation

Here we show the formulation of the constraints of ETFL. For clarity, we use different indexing sets, each referring to a specific object in the model. The definition of these, as well as that of the variables and the parameters, are detailed in Table 1. The formulation of the following equations and an explanation of the specific cases for RNA polymerase and ribosomes are discussed in details in the Methods section.

**Table 1:** Indices, variables, and parameters used in the formulation.

Index letter	Type	Refers to	Set or unit
$i$	Index	Metabolite	${\cal{I}}$
${{\rm{aa}}}_{i}$	Index	Amino acid	${\cal{A}}$
$j$	Index	Reaction/flux/enzyme	${\cal{J}}$
$l$	Index	Gene/peptide/mRNA	${\cal{L}}$
$s$	Index	Binary coefficient for growth discretization	${\cal{S}}=\left\{0..\lceil {{\log}}_{2}N\rceil \right\}$
$u$	Index	Binary coefficient for interpolation discretization	${\cal{U}}=\left\{0..N\right\}$
$\mu$	Variable	Growth rate	${{\rm{h}}}^{-1}$
${v}_{j}^{\pm }$	Variable	${j}^{{\rm{th}}}$ net positive/negative biochemical flux	${\rm{mmol}}.{{\rm{gDW}}}^{-1}.{{\rm{h}}}^{-1}$
${E}_{j}$	Variable	Concentration of the ${j}^{{\rm{th}}}$ enzyme	${\rm{mmol}}.{{\rm{gDW}}}^{-1}$
${F}_{l}$	Variable	Concentration of the ${l}^{{\rm{th}}}$ mRNA	${\rm{mmol}}.{{\rm{gDW}}}^{-1}$
${P}_{l}$	Variable	Concentration of the RNA polymerase assigned to the ${l}^{{\rm{th}}}$ mRNA	${\rm{mmol}}.{{\rm{gDW}}}^{-1}$
${R}_{l}$	Variable	Concentration of the ribosome assigned to the ${l}^{{\rm{th}}}$ peptide	${\rm{mmol}}.{{\rm{gDW}}}^{-1}$
${T}_{{{\rm{aa}}}_{i}}^{u}$	Variable	Concentration of the ${i}^{{\rm{th}}}$ uncharged tRNA	${\rm{mmol}}.{{\rm{gDW}}}^{-1}$
${T}_{{{\rm{aa}}}_{i}}^{c}$	Variable	Concentration of the ${i}^{{\rm{th}}}$ charged tRNA	${\rm{mmol}}.{{\rm{gDW}}}^{-1}$
${v}_{l}^{{\rm{tsl}}}$	Variable	Translation rate of the ${l}^{{\rm{th}}}$ gene	${\rm{mmol}}.{{\rm{gDW}}}^{-1}.{{\rm{h}}}^{-1}$
${v}_{l}^{{\rm{tcr}}}$	Variable	Transcription rate of the ${l}^{{\rm{th}}}$ gene	${\rm{mmol}}.{{\rm{gDW}}}^{-1}.{{\rm{h}}}^{-1}$
${v}_{j}^{{\rm{asm}}}$	Variable	Assembly rate of the ${j}^{\rm{th}}$ enzyme	${\rm{mmol}}.{{\rm{gDW}}}^{-1}.{{\rm{h}}}^{-1}$
${v}_{j}^{{\rm{deg}} }$	Variable	Degradation rate of the ${j}^{{\rm{th}}}$ enzyme	${\rm{mmol}}.{{\rm{gDW}}}^{-1}.{{\rm{h}}}^{-1}$
${v}_{l}^{{\rm{deg}} }$	Variable	Degradation rate of the ${l}^{{\rm{th}}}$ mRNA	${\rm{mmol}}.{{\rm{gDW}}}^{-1}.{{\rm{h}}}^{-1}$
${v}_{{{\rm{aa}}}_{i}}^{{\rm{charging}}}$	Variable	Charging rate of the tRNA associated to amino acid ${\rm{aa}}_i$	${\rm{mmol}}.{{\rm{gDW}}}^{-1}.{{\rm{h}}}^{-1}$
${k}_{{\rm{cat}}}^{j,\pm }$	Parameter	Forward/backward catalytic rate constant of the ${j}^{{\rm{th}}}$ net biochemical flux	${{\rm{h}}}^{-1}$
${k}_{{\rm{deg}} }^{j}$	Parameter	Degradation rate constant of the ${j}^{{\rm{th}}}$ enzyme	${{\rm{h}}}^{-1}$
${k}_{{\rm{deg}} }^{l}$	Parameter	Degradation rate constant of the ${l}^{{\rm{th}}}$ mRNA	${{\rm{h}}}^{-1}$
${\eta }_{l}^{j}$	Parameter	Stoichiometry of the ${l}^{{\rm{th}}}$ peptide in the ${j}^{{\rm{th}}}$ enzyme	$[\varnothing ]$
${\eta }_{{{\rm{aa}}}_{i}}^{l}$	Parameter	Stoichiometry of the amino acid ${\rm{aa}}_i$ in the ${l}^{{\rm{th}}}$ peptide	$[\varnothing ]$
${L}_{l}^{{\rm{aa}}}$	Parameter	Length in amino acids (aa) of the ${l}^{{\rm{th}}}$ peptide	${\rm{aa}}$
${L}_{l}^{{\rm{nt}}}$	Parameter	Length in nucleotides (nt) of the ${l}^{{\rm{th}}}$ mRNA	${\rm{b}}$
${L}_{{\rm{rib}}}^{{\rm{nt}}}$	Parameter	Ribosome footprint size on mRNA, in nucleotides	${\rm{b}}$
$\rho$	Parameter	Ribosome occupancy	$[\varnothing ]$
$\pi$	Parameter	RNA polymerase occupancy	$[\varnothing ]$

Metabolite mass balance

$$S\cdot v=0\qquad ({\mathrm{FBA}})$$

Catalytic constraints

$${v}_{j}^{+}-{k}_{{\rm{cat}}}^{j,+}{E}_{j}\le 0\qquad({{\rm{FC}}}_{{\rm{j}}})$$

$${v}_{j}^{-}-{k}_{{\rm{cat}}}^{j,-}{E}_{j}\le 0\qquad({{\rm{BC}}}_{{\rm{j}}})$$

Expression mass balance

$${v}_{l}^{{\rm{tsl}}}-\sum _{j\in {\mathcal{J}}}{\eta }_{l}^{j}\cdot {v}_{j}^{{\rm{asm}}}=0\qquad({{\rm{PB}}}_{{\rm{l}}})$$

$${v}_{{{\rm{rRNA}}}_{l}}^{{\rm{tcr}}}-{v}_{{\rm{rib}}}^{{\rm{asm}}}=0\qquad({{\rm{RB}}}_{{{\rm{rRNA}}}_{{\rm{l}}}})$$

$${v}_{j}^{{\rm{asm}}}-{v}_{j}^{{\mathrm{deg}} }-\mu * {E}_{j}=0\qquad({{\rm{EB}}}_{{\rm{j}}})$$

$${v}_{l}^{{\rm{tcr}}}-{v}_{l}^{{\mathrm{deg}} }-\mu * {F}_{l}=0\qquad({{\rm{MB}}}_{{\rm{l}}})$$

$$-{v}_{{{\rm{aa}}}_{i}}^{{\rm{charging}}}+\sum _{l\in {\mathcal{L}}}{\eta }_{{{\rm{aa}}}_{i}}^{l}\cdot {v}_{l}^{{\rm{tsl}}}-\mu * {T}_{{{\rm{aa}}}_{i}}^{u}=0\qquad({{\rm{TB}}}_{{{\rm{aa}}}_{{\rm{i}}}}^{{\rm{u}}})$$

$${v}_{{{\rm{aa}}}_{i}}^{{\rm{charging}}}-\sum _{l\in {\mathcal{L}}}{\eta }_{{{\rm{aa}}}_{i}}^{l}\cdot {v}_{l}^{{\rm{tsl}}}-\mu * {T}_{{{\rm{aa}}}_{i}}^{c}=0\qquad({{\rm{TB}}}_{{{\rm{aa}}}_{{\rm{i}}}}^{{\rm{c}}})$$

Degradation fluxes

$${v}_{j}^{{\mathrm{deg}} }-{k}_{{\mathrm{deg}} }^{j}\cdot {E}_{j}=0\qquad({{\rm{ED}}}_{{\rm{j}}})$$

$${v}_{l}^{{\mathrm{deg}} }-{k}_{{\mathrm{deg}} }^{l}\cdot {F}_{l}=0\qquad({{\rm{MD}}}_{{\rm{l}}})$$

Expression constraints

$${v}_{l}^{{\rm{tcr}}}-\frac{{k}_{{\rm{cat}}}^{{\rm{RNAP}}}}{{L}_{l}^{{\rm{nt}}}}{P}_{l}\le 0\qquad({\rm{TR}}{1}_{{\rm{l}}})$$

$${v}_{l}^{{\rm{tsl}}}-\frac{{k}_{{\rm{cat}}}^{{\rm{rib}}}}{{L}_{l}^{{\rm{aa}}}}{R}_{l}\le 0\qquad({\rm{TR}}{2}_{{\rm{l}}})$$

$${R}_{l}-\frac{{L}_{l}^{{\rm{nt}}}}{{L}_{{\rm{rib}}}^{{\rm{nt}}}}{F}_{l}\le 0\qquad({{\rm{EX}}}_{{\rm{l}}})$$

Total capacity

$$\sum _{l\in {\mathcal{L}}}{R}_{l}+{R}_{{\rm{F}}}-{E}_{{\rm{rib}}}=0\qquad({\rm{TC}}2)$$

$$\sum _{l\in {\mathcal{L}}}{P}_{l}+{P}_{{\rm{F}}}-{E}_{{\rm{RNAP}}}=0\qquad({\rm{TC}}1)$$

$${R}_{{\rm{F}}}-\left(1-\rho \right){E}_{{\rm{rib}}}=0\qquad({\rm{RR}})$$

$${P}_{{\rm{F}}}-\left(1-\pi \right){E}_{{\rm{RNAP}}}=0\qquad({\rm{PR}})$$

### Recovering the FBA problem

In the ETFL formulation, enzyme synthesis is driven by the coupling between FBA and EP through the catalytic constraints. To carry flux, the cell needs to produce enzymes which will also use the metabolic resources of the cell. If allocation constraints are enforced, the amount of protein and mRNA synthesized must meet predefined mass ratios for the problem to be feasible. Hence, the metabolic requirement terms for the expression machinery (amino acids and NTP) have been removed from the biomass reaction and are accounted for in the tRNA charging and transcription reactions. Thus, the FBA solutions can be recovered from the ETFL formulation by the following routine:

- Setting $$\forall j,\quad {k}_{{\rm{cat}}}^{j,\!\pm }=+\infty$$,
- Constraining $$\forall {{\rm{aa}}}_{i},\quad {v}_{{{\rm{aa}}}_{i}}^{{\rm{charging}}}={\eta }_{{{\rm{aa}}}_{i}}^{{v}_{{\rm{biomass}}}}\cdot \mu$$,
- Constraining $$\forall {{\rm{NTP}}}_{i},\quad {\sum }_{l\in {\mathcal{L}}}{v}_{{{\rm{NTP}}}_{i}}^{{{\rm{tcr}}}_{l}}={\eta }_{{{\rm{NTP}}}_{i}}^{{v}_{{\rm{biomass}}}}\cdot \mu$$,
- If applicable, relaxing the allocation constraints,
- If applicable, relaxing the thermodynamic coupling constraints.

### Application: *E. coli* genome-scale model iJO1366

iJO366^12^ is a well-curated and well-studied GEM of *E. coli* that is closely related to the GEM used in developing both ME-models iOL1650-ME^7^ and iJL1678b-ME^8^. In addition, this model has been extensively applied in the literature, and is aligned with a variety of data sets that can be used for data integration. We wanted to subject the model to classical studies that would highlight the power of ETFL, particularly as pertains to proteome-limited growth, macromolecule concentration variability analysis, and gene knockout studies. We also wanted to assess the sensitivity of the model with respect to the presence of thermodynamic constraints, as well as growth-dependent parameters.

Thus, we first experimented with four different models using ETFL with or without thermodynamic constraints and growth-dependent protein/RNA/DNA allocation following Table 2 as reported by Neidhardt et al.^11^. The following Table 2 details the nomenclature used to refer to these different models. The features of the most constrained model containing both thermodynamic and growth-dependent parameters, vETFL, are detailed in Table 3. These four models were optimized for maximal growth at increasing glucose uptake rates to assess their behavior with respect to excess substrate, which will show the non-linearity of the relationship between growth and glucose uptake at high-uptake rates. A plateau in the growth rate was expected, which indicates a proteome-limited phenotype that cannot be observed with FBA. We also subsequently subject vETFL to a variability analysis and gene essentiality analysis, which will, respectively, show us the flexibility of the model and its accuracy in predicting gene knockout behavior.

**Table 2:** Nomenclature of the models used in the study of *E. coli* iJO1366.

	Growth-independent parameters	Growth-dependent parameters
(−) thermodynamics	EFL	vEFL
(+) thermodynamics	ETFL	vETFL

EFL stands for Expression and FLuxes, ETFL for Expression, Thermodynamics, and FLuxes, and the v- prefix indicates the inclusion of growth-dependent parameters (see the section Discretization of mRNA and enzyme content in Methods.)

**Table 3:** Properties of the vETFL model generated from iJO1366.

Growth upper bound $\overline{\mu }$	3.5 h^−1^
Number of bins, $N$	128
Resolution, $\frac {\overline{\mu }}{N}$	0.0273 h^−1^
Number of constraints	68,304
Number of variables	49,207
Number of species	3240
– Metabolites	1806
– Peptides	1431
– rRNA	3
Number of enzymes	562
Number of reactions	8023
– Metabolic	1543
– Transport	733
– Exchange flux	330
– Transcription	1431
– Translation	1431
– Complexation	562
– Degradation	1993
Number of metabolites, ${\Delta }_{f}{G}^{{\prime} o}$	1737
Number of reactions, ${\Delta }_{r}{G}^{{\prime} o}$	1787
Percent of metabolites, ${\Delta }_{f}{G}^{{\prime} o}$	93.9%
Percent of reactions, ${\Delta }_{r}{G}^{{\prime} o}$	68.6%

### Growth rate prediction

To study the behavior of the model at different carbon uptake rates, we simulated growth on a minimal medium with only glucose as a carbon source, unlimited oxygen, and some essential inorganic compounds. This would allow us to show that at a higher carbon uptake, the model would predict a limited growth—unlike FBA that would predict an unlimited linear increase.

**Fig. 1:**
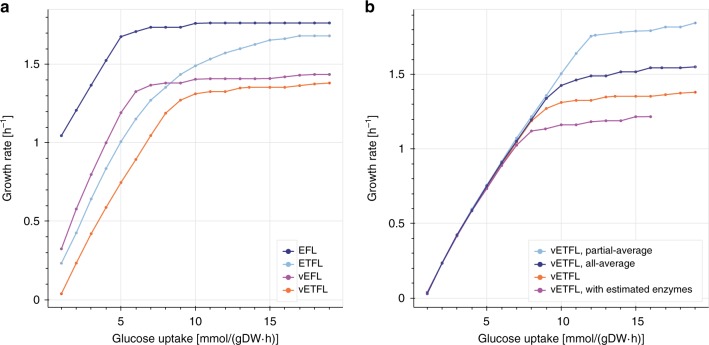
Growth rate with respect to glucose uptake for differently constrained models in the ETFL framework. Legend in the same order as the height of the right-most point of each curve in each figure. **a** Growth rate predictions using the EFL, ETFL, vEFL, vETFL models (dark blue, light blue, purple, orange); **b** growth rate predictions accounting for missing enzymes using vETFL (orange) and models (i)–(iii) (purple, dark blue, light blue) representing different initial enzyme assumptions, with ${k}_{{\rm{cat}}}$ values obtained from vETFL or ${k}_{{\rm{cat}}}=172\ {{\rm{h}}}^{-1}$, and with/without inferred enzymes.

 Figure 1 shows the predicted growth rate of the different (v)E(T)FL models described in Table 2 with respect to the glucose uptake of the cell. As expected and in contrast to current FBA models, all four models plateau after a certain uptake rate, which indicates a proteome-limited phenotype due to the limited capacity of the cells to make more enzymes to metabolize the glucose. As discussed for the ME-models^7^ and GECKO^5^ formulations, within the context of models accounting for protein usage, this is caused by (i) the protein burden necessary to metabolize higher fluxes; (ii) the increased demand in protein synthesis at higher growth rates; and (iii) for the models with allocation constraints, the allowed protein and RNA mass ratio. We can see that models featuring protein, RNA, and DNA allocation constraints (vE[T]FL) consistently predict a lower growth rate than models without allocation constraints. This is expected, as the data we input requires additional proteins and mRNA to account for non-metabolism-related macromolecules. Models featuring thermodynamic constraints ([v]ETFL) also predict a lower growth rate, consistent with the fact that thermodynamics constrain the model to valid solutions whose flux is in the subspace of the FBA feasible space. The most constrained model (vETFL) consequently has the lowest growth rate at any glucose uptake. This is in accordance with published TFA results that eliminated biologically infeasible flux profiles yielding non-realistic higher growth rates^2^.

We summarize the constraint matrix of the EP of vETFL in Supplementary Table 1, where each line represents a type of constraint and each column represents a type of variable. The blocks of the matrix that are nonzero are colored, and these blocks directly reflect the involvement of the constrained variables.

### Modeling missing enzymes

Although we initially focused on including only enzymes for which we had all the necessary information (catalytic rate and peptide constitution), we wanted to assess the robustness of our model when the missing enzymes were modeled as well as check our model’s sensitivity to changes in the catalytic rate constants. Thus, we additionally built three more models, based on vETFL, with the following properties: (i) all the missing enzymes were estimated by averaging the properties of the known enzymes based on the curation for the vETFL iJO1366 (333 amino acids long, average ${k}_{{\rm{cat}}}^{\pm }=172\ {{\rm{s}}}^{-1}$); (ii) all the enzymes (including the missing enzymes) but the ribosome, RNA polymerase, and ATP synthase were assumed to have an average catalytic rate constant ${k}_{{\rm{cat}}}^{\pm }=172\ {{\rm{s}}}^{-1}$; and, for comparison purposes, (iii) all the known enzymes of vETFL except for the ribosome, RNA polymerase, and ATP synthase were assumed to have an average catalytic rate constant of ${k}_{{\rm{cat}}}^{\pm }=172\ {{\rm{s}}}^{-1}$. For clarity, we will refer to these models as (i) the model with estimated enzymes; (ii) the all-average model; and (iii) the partial-average model. The ribosome, RNA polymerase, and ATP synthase were not modified, as their catalytic rates directly and strongly affect the growth of the organism. Any drastic change in these would make changes related to other enzymes negligible in comparison.

 Figure 1b shows a comparison of the growth prediction for the model with estimated enzymes (purple), all-average model (dark blue), and partial-average (light blue) models designed to account for the missing enzymes. For a better comparison, we also reproduce the vETFL results in orange on the same graph. The partial-average model (light blue) shows a higher predicted growth than the original vETFL model (orange). This implies that limiting enzymes in the original vETFL model have a ${k}_{{\rm{cat}}}$ parameter lower than the average value of ${k}_{{\rm{cat}}}^{\pm }=172\ {{\rm{s}}}^{-1}$. Both models featuring inferred enzymes, the all-average model (dark blue) and the model with estimated enzymes (purple) show, at a given uptake, a lower growth rate than their counterpart, respectively, the partial-average model (light blue) and the original vETFL (orange). This is expected as fluxes which previously had no enzymes assigned in vETFL are now subject to catalytic constraints, and thus the models are more constrained. In addition, we observe that the model with estimated enzymes (purple) is also below the all-average model (dark blue). Similarly to vETFL and the partial-average model, this shows that the limiting enzymes in the model with estimated enzymes have a ${k}_{{\rm{cat}}}$ parameter lower than the average value. Finally, we observe that the differences between these four models only appear at glucose uptake rates higher than $\approx \!6\ {{\rm{mmol}}}_{{\rm{glc}}}.{{\rm{DW}}}^{-1}.{{\rm{h}}}^{-1}$, when the problem switches from being stoichiometry-limited to proteome-limited. Thus, this experiment illustrates the robustness of the formulation in predicting growth-limited phenotypes, but also the importance of well-curated catalytic rate constants for modeling organisms grown in proteome-limited regimens.

These results demonstrate the capability of ETFL to predict different phenotypes depending on growth rate. ETFL is also amenable to hypothesis testing, as evidenced using the models that estimate the missing enzymes. In particular, we showed with ETFL that an uptake increase does not yield a proportional growth rate increase as with FBA and that ETFL provides a maximal uptake rate that is unmodeled in FBA, thus more effectively modeling growth-dependent biomass yield in *E. coli*. This allows for more realistic predictions for phenotypes that are limited by the expression capabilities of the cell as well as captures the variability of the biomass composition in different growth regimens.

### Variability analysis

It is also possible to subject the model to a range of variability analyses. These are routinely used in FBA to assess the flexibility of the system and in TFA to find the ranges of allowed metabolite concentrations. In particular, we studied the number of bidirectional reactions in the system. Bidirectional reactions are reactions whose net flux can be either positive or negative. They are an indicator of the flexibility of the system. One of the main results of TFA was to replace ad hoc assumptions on the directionality of the reactions by thermodynamically-based directionality. We show that adding enzymatic constraints with ETFL also reduces the number of bidirectional reactions. The initial iJO1366 formulation with ad hoc directionality assumptions shows 112 bidirectional reactions in FBA, under the constraint of a specific growth rate of $0.79\ {{\rm{h}}}^{-1}$ (TFA prediction). Once TVA is performed on the themodynamics-enabled model of iJO1366, the number of bidirectional reactions drops to 88. Finally, after the addition of catalytic constraints, this number is reduced to 49 in the vETFL model.

**Fig. 2:**
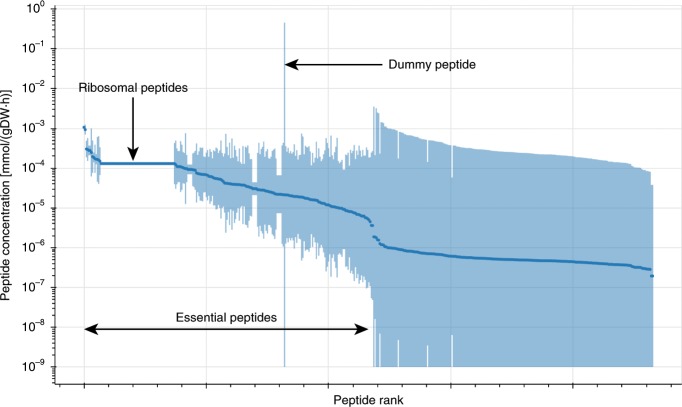
Concentration variability of peptide species, sorted by average peptide concentration (darker disc). Lower bounds that were 0 were cut off at concentrations of ${10}^{-9} \, {\mathrm{mmol.gDW}}^{-1}$. The horizontal line on the left side of the figure represents ribosomal peptides, which is narrow due to their instrumental role in making the tightly constrained amount of protein in the cell at a given growth rate. The vertical line in the middle represents the dummy peptide, which accounts for unmodeled peptides (non-metabolic proteins and enzymes with missing information) and therefore is used by the solver as a slack.

We can extend the use of variability analyses in ETFL to explore the allowed proteome and transcriptome. For example, we measured the admissible extreme concentrations of each peptide in aerobic growth conditions as described by McCloskey et al.^13^ by performing a variability analysis on the enzyme concentration variables. Figure 2 depicts the admissible peptide concentration upper and lower bounds, sorted by average, for vETFL with a glucose uptake set to $12.5\ {\rm{mmol}}.{{\rm{gDW}}}^{-1}.{{\rm{h}}}^{-1}$, which yields a proteome-limited phenotype, according to our results in Fig. 1a. It is important to note that all peptides with a nonzero minimal concentration (most of the left of the figure) are, by definition, essential peptides. These are always present at this uptake rate and are hence necessary for the cell to grow at an optimum growth rate. The same study can be performed for mRNA concentrations or even metabolite log-concentrations for models with thermodynamics. This type of study is useful for comparing how the model performs in relation to actual proteomics, transcriptomics, or metabolomics data. The method for running these other types of variability analyses is exactly the same—only the variables subject to the variability analysis are changed.

A specific usage of a variability analysis is the study of the allowed proteome (resp. transcriptome) that is done by performing a variability analysis on the enzyme (mRNA) concentration variables. This type of study can, for instance, be compared with transcriptomics to check if the expression profile of an engineered strain corresponds to what is expected in its corresponding model. A way to visualize the average allowed proteome (transcriptome) is to use the average value of the variability of each enzyme (mRNA) concentration as a feasible observation. Due to the convexity of the solution space, it is a solution to the problem. This observation is then plotted on a finite area, which can be done using the online software Proteomaps^14,15^. This method and software are often used by biologists to represent protein abundances in the cell, and using the data from ETFL, we can generate similar comparative graphs that can help biologists analyze the variability in the different concentration variables using a visualization they are familiar with.

**Fig. 3:**
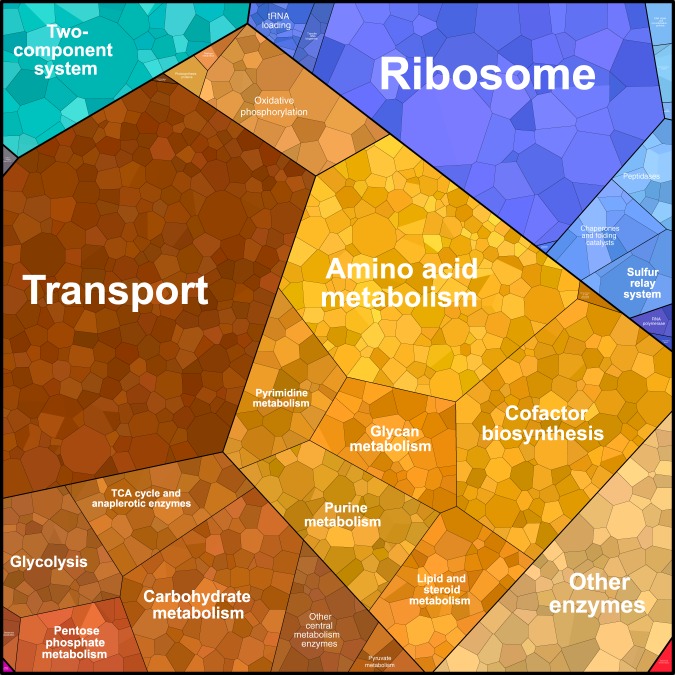
Voronoi map of the predicted abundances of mRNAs. Each colored patch represents a different mRNA, with its area in proportion to its relative abundance. Genes can be clustered using KEGG Gene Ontology (GO) terms. Colors indicate the clustering.

 Figure 3 is an example of such a representation, graphed using the mRNA concentrations corresponding to the solution represented by the darker dots in Fig. 2 as an input. In this figure, mRNAs are clustered using KEGG Gene Ontology (GO) annotations. GO annotations form a tree describing the physiological role of genes, ranging from the least specific (e.g. general metabolism) to most specific (e.g. araH gene). The area of each (sub)cluster is proportional to the relative abundance of each (sub)group of mRNAs.

We used the mean of the variability analysis as the observation rather than a single optimal solution because the optimality principle in LP only guarantees a unique global optimum value and not a unique optimal solution. Moreover, solver heuristics give sparse and extreme results (corners of the explored simplex), which do not accurately represent the full extent of the considered solution space.

### Essentiality analysis

The ETFL framework can also analyze the essentiality of specific genes by performing single gene knockouts. The growth of models with knocked-out genes can then be compared with the experimental data to assess the quality of the model as a validation.

**Fig. 4:**
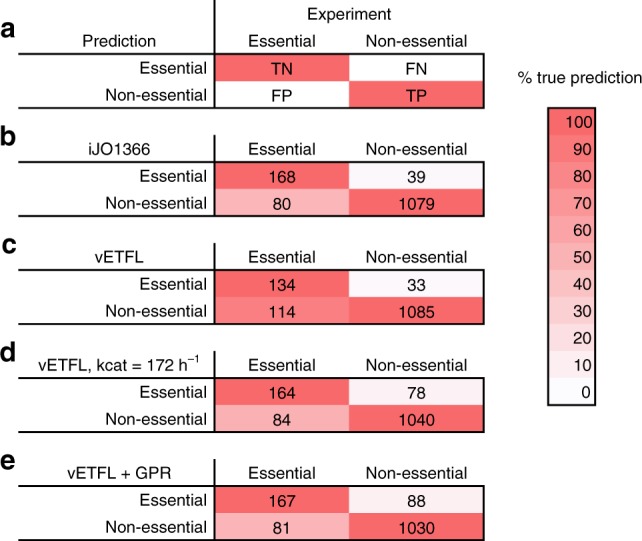
Confusion matrices for gene essentiality studies. **a** Conventions from Orth et al.^12^ for gene essentiality. TN is true negative. FN is false negative. FP is false positive. TP is true positive. The color shading represents how good the classification is. Perfect classification should have a strict red first diagonal, as shown on this example. **b** Gene essentiality prediction for the FBA model iJO1366, yielding a Matthew's correlation coefficient (MCC) of 0.69. **c** Gene essentiality prediction for the vETFL model, yielding a MCC of 0.60. **d** Gene essentiality prediction for the vETFL model with estimated enzymes with all ${k}_{{\rm{cat}}}=172\ {{\rm{h}}}^{-1}$, yielding a MCC of 0.59. **e** Gene essentiality prediction for the vETFL model, where genes without enzyme assignment were tested using gene to protein to reaction (GPR) associations from the iJO1366 model, yielding a MCC of 0.59.

We performed a gene essentiality analysis using in ETFL and compared it with the results reported in the publication of iJO1366 by Orth et al.^12^. We use the Matthew’s correlation coefficient (MCC) as a metric for the quality of the prediction, which is preferred over accuracy as it is not sensitive to the imbalance between the number of essential genes and non-essential genes. The MCC reads like a usual correlation coefficient, with 1 being a perfect correlation, −1 perfect anti-correlation, and 0 no correlation. We used the essentiality data and conventions given in the Supplementary Material of Orth et al.^12^, as explained in Fig. 4a, b. The results are presented in Fig. 4c, d, e, respectively, for vETFL, the model with estimated enzymes, and vETFL supplemented by iJO1366's Gene-Protein association Rules (GPRs).

Compared with iJO1366, we observe that ETFL predicts fewer false negatives (experimentally non-essential genes predicted as essential), However, ETFL also presents more false positives (experimentally essential genes predicted as non-essential). This indicates that ETFL is less constrained than iJO1366—the cell has more genetic alternatives for growth. This is an artificial effect that stems from the missing enzyme data.

As we add more enzyme information to the model, the false positive rate decreases. This is verified by Fig. 4d, where the addition of enzymes with average characteristics decreased the false positive rate. For comparison purposes, in Fig. 4e, we also computed the gene essentiality using iJO1366 gene to protein to reaction (GPR) associations for the genes which did not have an associated enzyme because of missing data. We show that the false positive rate decreases as well.

A detailed interpretation of the differences between gene knockout in ETFL and FBA is discussed in the Methods section. The Supplementary Data provide more insights on the mismatched between ETFL essentiality results and iJO1366 essentiality results, and indeed shows that $87 \%$ of the mismatches are attributed to reactions without enzymatic data. A significant fraction of mismatches ($54 \%$) come from the subsystems for the biosynthesis of lipids and cell envelope elements.

### Sampling

Sampling the feasible solution space of FBA is a common way to study solution robustness and variability. Since there are often multiple FBA solutions at the optimal objective value, representative solutions are often sought, and sampling is one way to obtain them. However, because ETFL contains integer variables, it is not compatible with traditional sampling methods in its current formulation. It is possible, though, to make the model convex, and hence amenable to sampling, by fixing the integers to their values at a given growth rate and, if applicable, TFA directionality. This will block the flux directions (if TFA is performed) as well as the growth-dependent parameters. The resulting model is then solely linear, and sampling can be performed with traditional techniques, such as artificially centered hit and run (ACHR)^16^, gpSampler^17^, or optGpSampler^18^. Once it has converged, a sampling should provide a better representation of the center of the solution space than the mean of the variability analysis.

### Performance

**Fig. 5:**
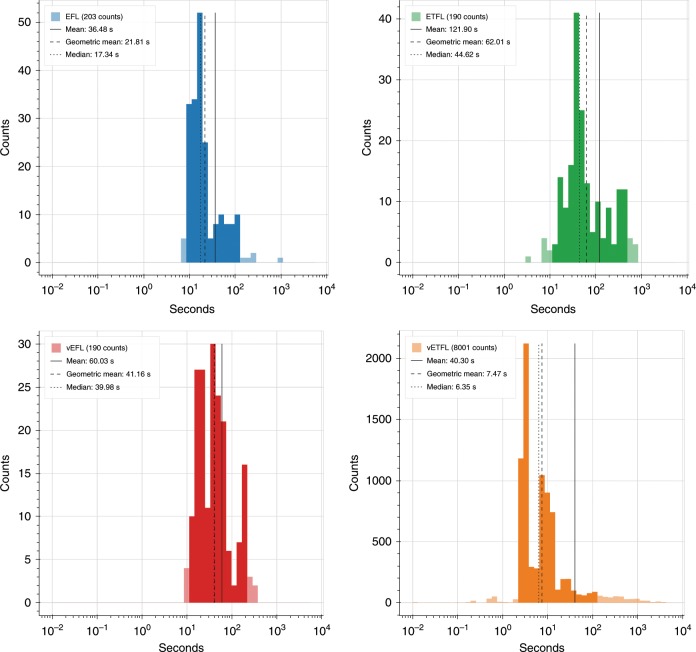
Histograms displaying the distribution of solving times of each type of model during the data generation for this study. The darker area represents data between the 5th and 95th percentiles.

For robustly reporting solution times of ETFL, we logged solving times each time a model was optimized during the redaction of this article. In that respect, some observations are the result of iterated optimizations, others from different optimization problems. In particular, variability and gene essentiality analyses require thousands of optimizations. We aggregated the solution times report the corresponding histograms, by model type, in Fig. 5. We measured the following metrics of the performance data: (i) arithmetic mean, (ii) geometric mean, and (iii) median. Although the distributions are not log-normal, it is common to report the geometric mean as a measure of the center of the distribution for comparison with other software^19,20^, as it is more robust to outliers than the arithmetic mean and more sensitive to unevenness than the median.

Using well-established MILP solvers (CPLEX^21^, Gurobi^22^), we report a geometric mean solution time of 7.47 s for vETFL, with 95% of the problems solved in <100 s on the test hardware. This is three orders of magnitude better than the reported solution time for O’Brien et al.^7^ (6 h – $2\,\times 1{0}^{4}$ s) and between one and two orders of magnitude better than the reported solution time for Lloyd et al. using cobraME^8^ (10 min – $6\,\times 1{0}^{2}$ s). It is worth noting that these vETFL optimizations also include thermodynamics constraints, which are absent of the other two formulations.

It is also important to state that although cobraME has an improved solution time over the original ME-model formulation, the formulation trades inequalities in the expression problem for equalities, and hence disregards a whole (non-growth optimal) part of the solution space that might contain physiological phenotypes. In particular, catalytic constraints become equalities, and the flux carried by reactions is set to be proportional to the amount of available enzyme instead of being upper-bounded by it. This gives less flexibility to the cell and prevents the representation of transient phenotypes. As an example, a cell that has been growing on a carbon source (e.g. glucose) will have a proteome suited to utilize this carbon source. However, once exhausted, it will need to reallocate its proteome to a new carbon source (e.g. lactose). In this transient state, some enzymes related to the first carbon source metabolism (e.g. glucose transporters) will carry no flux. In this case, cobraME would predict no flux, and also no enzyme concentration. In constrast, ETFL would allow for non-utilized enzymes and avoids such trade-offs, which is also crucial for accurately integrating proteomics data.

Such performance enhancements allow studies that would have been excessively time consuming using prior ME-model formulations. We show in Table 4 a list of typical completion times for common studies thats require multiple optimizations to be carried out.

**Table 4:** Characteristic completion run times for several types of studies in the vETFL study of iJO1366.

Study type (vETFL)	vETFL characteristic run time (h)
Growth curve (Fig. 1)	1
Enzyme VA	1.5
mRNA VA	2–3
Gene essentiality	10
50-points dETFL (see Dynamic ETFL Method)	1

Finally, ETFL relies on solver-specific MILP algorithms and heuristics, which also means that great variability in performances can be observed depending on the solver parameters. We provide tuned presets for different tasks (gene knockout, variability analysis, growth maximization) with the package, and recommend that users run their own solver tuning if long run times are observed. We witnessed an up to $10\! \times$ increase in performance using such tuning.

### Adaptation of FBA-based methods to ETFL

The ETFL formulation is amenable to further kinds of analyses. Leveraging both the explicit expression constraints and the MILP nature of the problem, we present several possibilities for future studies using ETFL:

### Growth-dependent parameters

It has been reported that several other parameters, such as the ribosome transcription rate constant ${k}_{{\rm{rib}}}$, are growth dependent^11^. Although such dependency is not taken into account in the presented results, it is possible to account for this by (i) discretizing ${k}_{{\rm{rib}}}$ following the method used to discretize the mRNA and protein content of the cell, and (ii) using Petersen’s linearization scheme (see the Methods section) on the product ${k}_{{\rm{rib}}}* {E}_{{\rm{rib}}}$. Other parameters that can be transformed in this way include, but are not limited to, the RNAP transcription rate constant ${k}_{trans}$, free ribosomes, and the RNAP ratios $\rho$ and $\pi$.

### Omics integration

Explicit mRNA and enzyme concentrations allow the direct integration of absolute or relative proteomics and transcriptomics by changing the bounds of the corresponding variables in the EP. An additional gauge constraint will be needed for the relative data. Previous transcriptomic integration methods, such as REMI^23^, iMAT^24^, GIMME^25^, or MINEA^26^, can also be adequately reformulated for ETFL. Metabolomics can still be integrated using TFA^2,3^.

### Minimization of adjustment

In the original paper, the hypothesis behind the Minimization of Metabolic Adjustment (MOMA) method is that the metabolic fluxes of an organism subject to a gene knockout show a minimal change compared with the metabolic fluxes of the wild-type organism^27^. The underlying hypothesis is that the enzyme distribution and assignments remain the same, except for the knocked-out gene. With ETFL, it is possible to directly compute a Minimization of Protein Adjustment (MOPA) by reformulating the objective function as such:

$$\min \ \sum _{j\in {\mathcal{J}}}\left|\left| {E}_{j}-{E}_{j}^{0}\right|\right|_{p},\quad p\in \left\{0,1\right\}\qquad({\rm{MOPA}})$$

where $| | \cdot | {| }_{p}$ is either the Manhattan norm ($p=1$, ${\ell }_{1}$-norm) or the Euclidean norm ($p=2$, ${\ell }_{2}$-norm), which will require a MIQP solver. In the same fashion, it is also possible to formulate a (weighted) Minimization of mRNA Adjustment (MORA) or even a Minimization of eXpression Adjustment (MOXA) using the following formulations:

$$\min \ \sum _{l\in {\mathcal{L}}}\left|\left| {F}_{l}-{F}_{l}^{0}\right|\right|_{p},\quad p\in \left\{0,1\right\}\qquad ({\rm{MORA}})$$

$$	\min \ \theta \cdot \sum _{j\in {\mathcal{J}}}\left|\left| {E}_{j}-{E}_{j}^{0}\right|\right|_{p}+\, (1-\theta )\cdot \sum _{l\in {\mathcal{L}}}\left|\left| {F}_{l}-{F}_{l}^{0}\right|\right|_{p}, \\ 	\quad\qquad\quad p\in \left\{0,1\right\},\theta \in \left[1,2\right].\qquad ({\rm{MOXA}})$$

### Parsimonious analysis

Parsimonious FBA (pFBA)^17^ was developed to address the high fluxes of some of the solutions given by FBA. Although this concern is addressed in ETFL by the combined actions of the EP and thermodynamics, pFBA can be adapted to ETFL to study an organism under parsimonious constraints. For example, it is possible to reformulate it into a parsimonious expression problem to find the minimal expression level required to meet a growth target using objective functions similar to (MOPA), (MORA), and (MOXA). It is also possible to turn the problem around to consider the allowed enzyme amounts under minimal flux constraint obtained by pFBA to assess the metabolic flexibility of an organism.

### Dynamic ETFL (dETFL)

Dynamic FBA (dFBA)^28^ is a method that uses FBA to predict the dynamics of a biological system represented with a stoichiometric model. In its original static optimization approach (SOA) formulation, a FBA problem is solved at each time step. The value of boundary fluxes of the FBA problem are updated at each iteration with values produced with a kinetic law, such as Michaelis–Menten glucose uptake and oxygen diffusion. Because ETFL allows direct access to enzyme concentrations, it is possible to use the latter to reformulate dFBA in its SOA. The original SOA approach uses ad hoc constraints on the absolute flux change at each time step. However, in ETFL, it is possible to bound flux changes indirectly by bounding enzyme and mRNA concentration changes in the EP. Effectively, this approach allows the movement from ad hoc constraints to physiological constraints.

### Use in kinetic frameworks

Often, kinetic frameworks require a reference flux distribution as an input. ETFL can provide such a distribution, with an increased accuracy as compared with FBA.

### Building an ETFL ME-model for other organisms

Building an ETFL model from a genome-scale model follows a detailed procedure, for which a SOP is provided in the Supplementary Note 2. In this procedure, it is the quality of the input data that will determine the accuracy of the model. A well-curated, elementally balanced model is a critical prerequisite. Since ETFL is essentially adding constraints to the FBA problem, it is important as well to ensure the feasibility of the initial model.

In ETFL, and ME-models in general, catalytic constraints are what links the metabolism to the expression problem. Because of this, the accuracy of the ETFL reconstruction is also heavily dependent on the quality of the catalytic rate constants ${k}_{{\rm{cat}}}^{j}$. Such information is not always easily accessible. Hence, we recommend to at least manually curate the catalytic rate constants of the key parts of metabolism, namely (i) ATP synthase, (ii) RNA polymerase, and (iii) ribosome. We also advise to pay attention to the pathways of the main carbon source metabolism, as small catalytic rate constants can heavily throttle the rest of the metabolism. For missing catalytic rate constants, a placeholder value can be used. O’Brien et al.^7^ used ${k}_{{\rm{cat}}}^{j}=65\ {{\rm{s}}}^{-1}$, which is close to the median of the values used in this study. In our comparison with inferred enzymes, we used ${k}_{{\rm{cat}}}^{j}=172\ {{\rm{s}}}^{-1}$, which is the arithmetic mean of the data we gathered.

Another key component for catalytically constraining the model is to have quality enzyme composition information. Indeed, marking an enzyme as a monomer instead of a dimer halves its synthesis cost. A good source for this information is MetaCyc^29^, and literature. As explained in the previous paragraph, special attention should be given to the ATP synthase, the RNA polymerase, the ribosome, and the enzymes of the main carbon pathway. Macromolecule degradation rates are less critical and can be averaged. Growth-dependent protein, RNA, and DNA ratios drastically improve the quality of the model, as they allow to account for the expression activity that is related to non-metabolic processes.

In the construction of a model for another organism, approximating parameters based on values from an *E. coli* model should be done with care. Similarly to gap filling and the use of template reactions, conserving parameters across close species is helpful; however, conserving parameters across a large phylogenetic distance is erroneous. An example is the ribosome translation rate, which can vary by one order of magnitude between *S. cerevisiae* and *E. coli*.

Finally, great care should be taken with respect to the units. Different conventions are used across sources. Parameters for which this has been observed include catalytic rate constants, molecular weights, and concentrations.

## Conclusions

ETFL is a framework which implements expression and thermodynamic formalism using mainstream double-precision MILP solvers. This could not be previously accomplished using state-of-the-art ME-models, which use specialized quad-precision solvers and do not support integer variables. The formalism itself is based on the explicit and direct relationship with the underlying biochemistry and provides a way to incorporate growth-dependent variables using MILP linearization techniques. These new growth-dependent variables provide a finer modeling of expression because they consider phenotypic differences in different growth regimens, which are key for accurate modeling. ETFL can also compute explicit mRNA and enzyme concentrations as well as perform direct -omics data integration. In this, ETFL complements and extends FBA capabilities by using explicit relationships in lieu of the typical assumptions on the relationships between the transcriptome, proteome, and fluxome. This explicit accounting of expression mechanisms provides a finer level of control and a more relevant prediction of gene-editing outcomes. ETFL is robust to missing data, as missing enzymes and their composition can be approximated using average enzyme characteristics. Because of this and its operational similarity with classic FBA-related analyses, ETFL can be efficiently integrated in standard model-based pipelines. For this intent, we provide in the Supplementary Note 2 a standardized procedure to produce ETFL models from genome-scale models. For example, metagenome-based genome-scale reconstructions such as published by Magnúsdóttir et al.^1^ can be directly fed to the framework to generate models for each of the 773 bacteria they identified. Integration with platforms like KBase^30^ can also be envisioned to automatically draft ETFL reconstructions parametrized by curated organism-specific data. In a more general way, ETFL can assess the allowed expression profiles of any biological system amenable to genome-scale modeling, such as in the metabolic engineering of biocatalysts, microbial communities, drug design, or personalized medicine.

## Methods

### Preliminaries, conventions, and notations

The mass balances of the macromolecules in ME-models are written with respect to their concentration variables. If we assume the cell is growing at a specific growth rate $\mu$, we must assume that the volume of cell within which the mass balance is considered varies.

The mass balance of a macromolecule $G$ will be written:

5 $$\frac{{\mathrm{d}}{m}_{G}}{{\mathrm{d}}t}={C}_{G}\frac{{\mathrm{d}}{V}_{c}}{{\mathrm{d}}t}+{V}_{c}\frac{{\mathrm{d}}{C}_{G}}{{\mathrm{d}}t},$$

6 $$\qquad ={v}_{G}^{{\rm{syn}}}\cdot {V}_{c}-{v}_{G}^{{\mathrm{deg}} }\cdot {V}_{c},$$

where ${C}_{G}$ is the concentration of the macromolecule ${C}_{G}$ in the cellular volume ${V}_{c}$, for a total mass ${m}_{G}$ in the cell, produced at a rate ${v}_{G}^{{\rm{syn}}}$ and degraded at a rate ${v}_{G}^{{\mathrm{deg}}}$.

We next combine Eqs. (5) and (6) and divide by ${V}_{c}$ (necessarily nonzero) to write the time derivative of the concentration ${C}_{G}$:

7 $$\frac{{\mathrm{d}}{C}_{G}}{{\mathrm{d}}t}={v}_{G}^{{\rm{syn}}}-{v}_{G}^{{\mathrm{deg}} }-\frac{1}{{V}_{c}}\frac{{\mathrm{d}}{V}_{c}}{{\mathrm{d}}t}\cdot {C}_{G}.$$

By definition, $\frac{1}{{V}_{c}}\frac{{\mathrm{d}}{V}_{c}}{{\mathrm{d}}t}=\mu$ is the specific growth rate of the cell (under the assumption of constant cell density ${\rho }_{c}$), and the term $\mu \cdot {C}_{G}$ is called the dilution term, or ${v}_{G}^{{\rm{dil}}}$, as per Fredrickson’s work on formulating growth models^31^. It is a common assumption that the concentrations inside the cell remain time invariant (quasi-steady-state assumption), effectively yielding the constraint:

8 $${v}_{G}^{{\rm{syn}}}-{v}_{G}^{{\mathrm{deg}} }-\frac{1}{{V}_{c}}\frac{{\mathrm{d}}{V}_{c}}{{\mathrm{d}}t}\cdot {C}_{G}=0.$$

It is also understood from the formulation of the FBA that adding a new reaction to the system, such as:

9 $${v}_{j}:\qquad {\eta }_{A}^{j}A\to {\eta }_{B}^{j}B,$$

results in adding terms to the mass balances of $A$ and $B{\!\!}:$

10 $$\frac{{\mathrm{d}}\left[A\right]}{{\mathrm{d}}t}=\ldots -{\eta }_{A}^{j}\cdot {v}_{j},$$

11 $$\frac{{\mathrm{d}}\left[B\right]}{{\mathrm{d}}t}=\ldots +{\eta }_{B}^{j}\cdot {v}_{j}.$$

The further extension of this to reactions of *n* reactants to *m* products is trivial.

Several parameter values are taken from the BioNumbers database^32^. When used, we specify their identification number as well as the original source from which the value was reported. Finally, we will represent products between a parameter value and a variable by the symbol “$\cdot$” and products between two variables by the symbol “$*$”.

Hereafter, we propose a detailed top–down approach to formulate the constraints being built for ETFL, starting from the metabolite network and moving down to RNA synthesis. The general organization for each macromolecule is to write down its mass balance, apply assumptions, and then detail its synthesis and consumption mechanisms.

### Metabolites

From FBA, the mass–balance relationship for metabolites can be written as:

$$S\cdot v=0.\qquad ({\rm{FBA}})$$

For the rest of the formulation, it is necessary to split the net flux $v$ from each reaction into its forward net component and backward net component:

12 $${v}_{j}={v}_{j}^{+}-{v}_{j}^{-},\qquad {v}_{j}^{+},{v}_{j}^{-}\ge 0.$$

Biochemical reactions are catalyzed by enzymes. Each enzyme $({{\rm{Enz}}}_{j})$ of concentration ${E}_{j}$ can catalyze a flux ${v}_{j}$ subject to the enzyme capacity constraint, which is a function of its forward and backward catalytic rate constants ${k}_{{\rm{cat}}}^{j,+}$ and ${k}_{{\rm{cat}}}^{j,-}$:

13 $$0\le {v}_{j}^{+}\le {k}_{{\rm{cat}}}^{j,+}{E}_{j},$$

14 $$0\le {v}_{j}^{-}\le {k}_{{\rm{cat}}}^{j,-}{E}_{j},$$

$${v}_{j}^{+}-{k}_{{\rm{cat}}}^{j,+}{E}_{j}\le 0,\qquad({{\rm{FC}}}_{{\rm{j}}})$$

$${v}_{j}^{-}-{k}_{{\rm{cat}}}^{j,-}{E}_{j}\le 0.\qquad({{\rm{BC}}}_{{\rm{j}}})$$

The distinction between the bounds of the forward and backward net fluxes is important, as some enzymes have different catalytic activities, depending on the direction of the flux.

### General constraints for enzymes

Each enzyme ${{\rm{Enz}}}_{j}$ is represented by its total concentration, the variable ${E}_{j}$. It is subject to mass balance, which can be written:

15 $$\frac{{\mathrm{d}}}{{\mathrm{d}}t}{E}_{j}={v}_{j}^{{\rm{asm}}}-{v}_{j}^{{\mathrm{deg}} }-{v}_{j}^{{\rm{dil}}},$$

which reads under quasi-steady-state assumption (QSSA):

$${v}_{j}^{{\rm{asm}}}-{v}_{j}^{{\mathrm{deg}} }-\mu * {E}_{j}=0,\qquad ({{\rm{EB}}}_{{\rm{j}}})$$

where ${v}_{j}^{{\rm{asm}}}$ is the formation rate of the enzyme by the assembly of its constituent peptides, ${v}_{j}^{{\mathrm{deg}} }$ is the degradation rate, ${v}_{j}^{{\rm{dil}}}$ is the dilution rate, and $\mu$ is the growth rate of the cell. The formation rate of the enzyme describes the assembly of free peptides, hence it is necessary to add the peptide assembly reaction to the stoichiometric matrix:

16 $${v}_{j}^{{\rm{asm}}}:\qquad {\sum _{l\in {\mathcal{L}}}}{\eta }_{l}^{j}\cdot {{\rm{Pep}}}_{l}\to {{\rm{Enz}}}_{j},$$

where ${\eta }_{l}^{j}$ is the stoichiometric coefficient of peptide ${\mathrm{Pe}}{{\mathrm{p}}}_{l}$ for the formation of the complex of enzyme ${{\rm{Enz}}}_{j}$. This reaction is assumed to happen spontaneously by default.

We model the degradation reaction of the enzyme in the following manner:

17 $${v}_{j}^{{\mathrm{deg}} }:\quad {{\rm{Enz}}}_{j}+{L}_{j}^{{\rm{aa}}}\cdot {{\rm{H}}}_{2}{\rm{O}}\to {\sum _{{{\rm{aa}}}_{i}\in {\mathcal{A}}}}{\eta }_{{{\rm{aa}}}_{i}}^{j}\cdot {{\rm{aa}}}_{i},$$

where ${\eta }_{{{\rm{aa}}}_{i}}^{j}$ is the number of amino acids ${{\rm{aa}}}_{i}$ in the enzyme. It is obtained from the composition of the constituent peptides. For this degradation reaction, the rate is known:

$${v}_{j}^{{\mathrm{deg}} }-{k}_{{\mathrm{deg}} }^{j}\cdot {E}_{j}=0,\qquad ({{\rm{ED}}}_{{\rm{j}}})$$

where ${k}_{{\mathrm{deg}} }^{j}$ is the degradation rate constant of the enzyme. The reaction is added to the model, and the Eq. ${{\rm{ED}}}_{{\rm{j}}}$ is added as a constraint.

### Constraints specific to ribosomes

Like any other enzyme, ribosomes verify the mass balance:

$${v}_{{\rm{rib}}}^{{\rm{asm}}}-{v}_{{\rm{rib}}}^{{\mathrm{deg}} }-\mu * {E}_{{\rm{rib}}}=0\qquad ({EB}_{{\rm{rib}}}).$$

${E}_{{\rm{rib}}}$ denotes the total concentration of ribosomes in a cell. It accounts for ${R}_{l}$, the ribosomes assigned to the translation of ${{\rm{Pep}}}_{l}$, as well as the free ribosomes in the cell, ${R}_{{\rm{F}}}$.

The ribosome differs from other enzymes in that it takes ribosomal peptides ${{\rm{rPep}}}_{l}$, as well as ribosomal RNA ${{\rm{rRNA}}}_{l}$ for its assembly. Hence, its assembly reaction is:

18 $${v}_{{\rm{rib}}}^{{\rm{asm}}}:\qquad {\sum _{l\in {\mathcal{L}}}}{\eta }_{{{\rm{rPep}}}_{l}}^{{\rm{rib}}}\cdot {{\rm{rPep}}}_{l}+{\sum _{l\in {\mathcal{L}}}}{\eta }_{{{\rm{rRNA}}}_{l}}^{{\rm{rib}}}\cdot {{\rm{rRNA}}}_{l}\to {\rm{Rib}}.$$

As explained earlier, the stoichiometric coefficients ${\eta }_{\star }^{{\rm{rib}}}$ will appear in the mass balances of each of the compounds of the reaction. This reaction is also assumed to happen spontaneously by default.

When ribosomes are degraded, their constituting amino acids and ribonucleotides are recovered:

19 $${v}_{{\rm{rib}}}^{{\mathrm{deg}} }:\quad {\rm{Rib}}+{L}_{{\rm{rib}}}^{{\rm{aa}}}\cdot {{\rm{H}}}_{2}{\rm{O}}\to {\sum _{{{\rm{aa}}}_{i}\in {\mathcal{A}}}}{\eta }_{{{\rm{aa}}}_{i}}^{{\rm{rib}}}\cdot {{\rm{aa}}}_{i}+{\sum _{N\in {\rm{A,U,G,C}}}}{\eta }_{N}^{{\rm{rib}}}\cdot {\rm{NMP}}$$

The degradation rate is constrained in a manner similar to the constraint $({{\rm{ED}}}_{{\rm{j}}})$.

Finally, we can then write the total ribosome capacity constraint:

$$\sum _{l\in {\mathcal{L}}}{R}_{l}+{R}_{{\rm{F}}}-{E}_{{\rm{rib}}}=0.\qquad ({\rm{TC}}2)$$

If we know the ratio $\rho$ of occupied vs free ribosomes, we can enforce it:

$${R}_{{\rm{F}}}-\left(1-\rho \right){E}_{{\rm{rib}}}=0.\qquad ({\rm{RR}})$$

### Constraints specific to RNA polymerase

RNAP is an enzyme, and hence it also satisfies mass balance:

$${v}_{{\rm{RNAP}}}^{{\rm{asm}}}-{v}_{{\rm{RNAP}}}^{{\mathrm{deg}} }-\mu * {E}_{{\rm{RNAP}}}=0,\qquad ({EB}_{{\rm{RNAP}}})$$

where ${E}_{{\rm{RNAP}}}$ is the total amount of RNAP, which also accounts for free RNAP ${P}_{{\rm{F}}}$. Its synthesis and degradation follow equations similar to other enzymes:

$${v}_{{\rm{RNAP}}}^{{\rm{asm}}}-{v}_{{\rm{RNAP}}}^{{\mathrm{deg}} }-\mu * {E}_{{\rm{RNAP}}}=0,\qquad ({EB}_{{\rm{RNAP}}})$$

with the same conventions as in Eq. (${{\rm{EB}}}_{{\rm{j}}}$). As for a generic enzyme, RNAP is assembled from free peptides, which adds the peptide assembly reaction to the stoichiometric matrix:

20 $${v}_{{\rm{RNAP}}}^{{\rm{asm}}}:\qquad {\sum _{l\in {\mathcal{L}}}}{\eta }_{l}^{{\rm{RNAP}}}\cdot {{\rm{Pep}}}_{l}\to {\rm{RNAP}},$$

again with the same conventions as in the section General constraints for enzymes. This reaction is also assumed to happen spontaneously by default. The degradation reaction is also modeled similarly, with the same conventions:

21 $${v}_{{\rm{RNAP}}}^{{\mathrm{deg}} }:\quad {{\rm{Enz}}}_{j}+{L}_{j}^{{\rm{aa}}}\cdot {{\rm{H}}}_{2}{\rm{O}}\to {\sum _{{{\rm{aa}}}_{i}\in {\mathcal{A}}}}{\eta }_{{{\rm{aa}}}_{i}}^{j}\cdot {{\rm{aa}}}_{i}.$$

The degradation rate is constrained in a manner similar to the constraint $({{\rm{ED}}}_{{\rm{j}}})$.

In addition, the total capacity of RNAP follows a capacity constraint similar to that of ribosomes:

$$\sum _{l\in {\mathcal{L}}}{P}_{l}+{P}_{{\rm{F}}}-{E}_{{\rm{RNAP}}}=0.\qquad ({\rm{TC}}1)$$

As we did with the ribosomes, if we know the ratio of occupied RNAP, $\pi$, we can enforce it:

$${P}_{{\rm{F}}}-\left(1-\pi \right){E}_{{\rm{RNAP}}}=0.\qquad ({\rm{PR}})$$

### Constraints for peptides

The peptide concentrations obey the mass–balance equation:

22 $$\frac{d}{dt}{{\rm{Pep}}}_{l}={v}_{l}^{{\rm{tsl}}}-{\sum _{j\in {\mathcal{J}}}}{\eta }_{l}^{j}\cdot {v}_{j}^{{\rm{asm}}}-{v}_{l}^{{\mathrm{deg}} }-{v}_{l}^{{\rm{dil}}}.$$

We assume in the current model that the protein assembly rates are much faster than dilution and degradation, and thus simplify this mass balance to:

23 $$\frac{{\mathrm{d}}}{{\mathrm{d}}t}{{\rm{Pep}}}_{l}={v}_{l}^{{\rm{tsl}}}-{\sum _{j\in {\mathcal{J}}}}{\eta }_{l}^{j}\cdot {v}_{j}^{{\rm{asm}}},$$

which, under QSSA, can be written:

$${v}_{l}^{{\rm{tsl}}}-\sum _{j\in {\mathcal{J}}}{\eta }_{l}^{j}\cdot {v}_{j}^{{\rm{asm}}}=0.\qquad ({{\rm{PB}}}_{{\rm{l}}})$$

In this context, the peptides are treated just like regular metabolites in the system. This assumption in $({{\rm{PB}}}_{{\rm{l}}})$ can be relaxed without a loss of generality by introducing a dilution and a degradation term, thus introducing a bilinearity.

The synthesis of peptides consumes charged tRNAs, which are subsequently uncharged during the current peptide synthesis by a ribosome. The process consumes two GTPs and releases two GDPs and two Pi per amino acid:

24 $$\begin{array}{ll}{v}_{l}^{{\rm{tsl}}}:{\sum _{{{\rm{aa}}}_{i}\in {\mathcal{A}}}}{\eta }_{{{\rm{aa}}}_{i}}^{l}\cdot {{\rm{tRNA}}}_{{{\rm{aa}}}_{i}}^{{\rm{charged}}}+2{L}_{l}^{{\rm{aa}}}\cdot \left({\rm{GTP}}+{{\rm{H}}}_{2}{\rm{O}}\right)\hfill\\ \qquad\to {{\rm{Pep}}}_{l}+{\sum _{{{\rm{aa}}}_{i}\in {\mathcal{A}}}}{\eta }_{{{\rm{aa}}}_{i}}^{l}\cdot {{\rm{tRNA}}}_{{{\rm{aa}}}_{i}}^{{\rm{uncharged}}}+2{L}_{l}^{{\rm{aa}}}\cdot \left({\rm{GDP}}+{\rm{Pi}}+{{\rm{H}}}^{+}\right),\end{array}$$

where ${{\rm{aa}}}_{i}$ denotes the ${i}^{{\rm{th}}}$ amino acid, ${\eta }_{{{\rm{aa}}}_{i}}^{l}$ its stoichiometric coefficient (count) in the sequence of ${{\rm{Pep}}}_{l}$, ${{\rm{tRNA}}}_{{{\rm{aa}}}_{i}}^{* }$ the (un)charged tRNAs for each amino acid, and ${L}_{l}^{{\rm{aa}}}={\sum }_{{{\rm{aa}}}_{i}\in {\mathcal{A}}}{\eta }_{{{\rm{aa}}}_{i}}^{l}$ is the length of the amino acid sequence of ${{\rm{Pep}}}_{l}$.

As explained in the section Preliminaries, conventions, and notations, this reaction adds a supplementary term in the mass balances of the metabolites (GTP, GDP, Pi, ${{\rm{H}}}_{2}$O, ${{\rm{H}}}^{+}$), the peptide, and the tRNAs (see Constraints specific to tRNAs for the latter). This term is what connects the expression requirements to the metabolic network defined in the FBA.

The peptides are the product of a translation reaction that is catalyzed by a ribosome. As we did with the catalytic constraints for general biochemistry reactions, we can apply the ribosome maximum catalytic rate as an upper bound to its translation rate ${v}_{l}^{{\rm{tsl}}}$:

$${v}_{l}^{{\rm{tsl}}}-\frac{{k}_{{\rm{cat}}}^{{\rm{rib}}}}{{L}_{l}^{{\rm{aa}}}}{R}_{l}\le 0,\qquad({\rm{TR}}{2}_{{\rm{l}}})$$

where ${k}_{{\rm{cat}}}^{{\rm{rib}}}$ is the maximum ribosomal translation rate constant ($10-12{\rm{aa}}.{{\rm{s}}}^{-1}$ for *E. coli*, BioNumbers ID [BNID] 100059^33^), ${L}_{l}^{{\rm{aa}}}$ is the amino acid length of the peptide $l$, and ${R}_{l}$ is the concentration (in ${\rm{mmol}}.{{\rm{gDW}}}^{-1}$) of ribosomes assigned to the translation of this peptide. This way, the ratio ${R}_{l}/{{\rm{Pep}}}_{l}$ is effectively the number of ribosomes, or average polysome size, translating the peptide $l$.

### Constraints for mRNAs

During the translation, an mRNA is read to produce a peptide. mRNAs are subject the same mass–balance constraints:

$${v}_{l}^{{\rm{tcr}}}-{v}_{l}^{{\mathrm{deg}} }-\mu * {F}_{l}=0,\qquad ({{\rm{MB}}}_{{\rm{l}}})$$

where ${F}_{l}$ is the total concentration of the ${l}^{{\rm{th}}}$ mRNA $\left({\mathrm{mRNA}}_{l}\right)$, ${v}_{l}^{{\mathrm{deg}} }$ is its degradation rate, and ${v}_{l}^{{\rm{tcr}}}$ is its transcription (synthesis) rate. ${F}_{l}$ is variable that represents the concentration of $\left({\mathrm{RNA}}_{l}\right)$. The transcription reaction is modeled as follows:

25 $$\begin{array}{ll}{v}_{l}^{{\rm{tcr}}}:\,\, {\eta }_{A}^{l}\cdot {\mathrm{ATP}}+{\eta }_{U}^{l}\cdot {\mathrm{UTP}}+{\eta }_{C}^{l}\cdot {\mathrm{CTP}}+{\eta }_{G}^{l}\cdot {\mathrm{GTP}}\\ \to \left({\eta }_{A}^{l}+{\eta }_{U}^{l}+{\eta }_{C}^{l}+{\eta }_{G}^{l}\right) {\mathrm{PPi}}+ {\mathrm{mRNA}}_{l}.\end{array}$$

Again, the stoichiometric coefficients will appear in the mass balances of each of the metabolites and macromolecules involved. The transcription process is catalyzed by RNA polymerase (RNAP). For each transcription of mRNA, we can put an upper bound on the transcription rate ${v}_{l}^{{\rm{tcr}}}$ in the same way as for translation:

$${v}_{l}^{{\rm{tcr}}}-\frac{{k}_{{\rm{cat}}}^{{\rm{RNAP}}}}{{L}_{l}^{{\rm{nt}}}}{P}_{l}\le 0,\qquad ({\rm{TR}}{1}_{{\rm{l}}})$$

where ${L}_{l}^{{\rm{nt}}}$ is the length in nucleotides of the mRNA sequence, ${k}_{{\rm{cat}}}^{{\rm{RNAP}}}$ is the catalytic rate constant of RNAP ($85\,{\rm{nt}}.{{\rm{s}}}^{-1}$ for *E. coli*, BNID 100060^33^), and ${P}_{l}$ the concentration of RNAP assigned to the transcription of this mRNA.

We must also take into account the relationship between ribosome assignment and mRNA concentration. On each strand of ${{\rm{mRNA}}}_{l}$, there can be only a finite number ${\rho }_{l}$ of ribosomes translating at the same time. This number is given by the ratio of the footprint size of the ribosome ${L}_{{\rm{rib}}}^{{\rm{nt}}}$ and the length of the mRNA strand ${L}_{l}^{{\rm{nt}}}$. This effectively yields the number of ribosomes that can be present at the same time on a given mRNA strand:

26 $${\rho }_{l}=\frac{{L}_{l}^{{\rm{nt}}}}{{L}_{{\rm{rib}}}^{{\rm{nt}}}}.$$

For *E. coli*, ${L}_{{\rm{rib}}}^{{\rm{nt}}}$ is ~20 nm (BNID 102320^34^, 100121^35^), which amounts to ~60 base pairs (the length of a nucleotide is ~0.3 nm; BNID 103777^36^). From there we can get the additional constraint:

27 $${R}_{l}\le {\rho }_{l}\cdot {F}_{l},$$

$${R}_{l}-\frac{{L}_{l}^{{\rm{nt}}}}{{L}_{{\rm{rib}}}^{{\rm{nt}}}}{F}_{l}\le 0.\qquad ({{\rm{EX}}}_{{\rm{l}}})$$

We consider the following degradation reaction for mRNAs:

28 $${v}_{l}^{{\mathrm{deg}} }:\qquad {{\rm{mRNA}}}_{l}\to {\eta }_{A}^{l}\cdot {\rm{AMP}}+{\eta }_{U}^{l}\cdot {\rm{UMP}}+{\eta }_{C}^{l}\cdot {\rm{CMP}}+{\eta }_{G}^{l}\cdot {\rm{GMP}}.$$

And, again, we know the degradation rates:

$${v}_{l}^{{\mathrm{deg}} }-{k}_{{\mathrm{deg}} }^{l}\cdot {F}_{l}=0.\qquad ({{\rm{MD}}}_{{\rm{l}}})$$

### Constraints specific to rRNAs

rRNAs are used in the ribosome assembly reaction. According to the definition of ${v}_{{\rm{rib}}}^{{\rm{asm}}}$ in the section Constraints specific to ribosomes, their mass balance can be written:

29 $$\frac{{\mathrm{d}}}{{\mathrm{d}}t}\left[{{\rm{rRNA}}}_{l}\right]=0={v}_{{{\rm{rRNA}}}_{l}}^{{\rm{tcr}}}-{v}_{{\rm{rib}}}^{{\rm{asm}}}-{v}_{{{\rm{rRNA}}}_{l}}^{{\mathrm{deg}} }-{v}_{{{\rm{rRNA}}}_{l}}^{{\rm{dil}}}.$$

We neglect their dilution and degradation under the hypothesis that free rRNAs are scarce and stable^37^. Thus, their mass balance in the model reads:

$${v}_{{{\rm{rRNA}}}_{l}}^{{\rm{tcr}}}-{v}_{{\rm{rib}}}^{{\rm{asm}}}=0.\qquad ({{\rm{RB}}}_{{{\rm{rRNA}}}_{{\rm{l}}}})$$

The degradation reaction is the same as for mRNA, and is part of the total degradation of the ribosome.

### Constraints specific to tRNAs

Since tRNAs are relatively stable molecules^37^, we neglect their degradation. Let ${T}_{{{\rm{aa}}}_{i}}^{u}$ (resp. ${T}_{{{\rm{aa}}}_{i}}^{c}$) represent $\left[{{\rm{tRNA}}}_{{{\rm{aa}}}_{i}}^{{\rm{uncharged}}}\right]$ (resp. $\left[{{\rm{tRNA}}}_{{{\rm{aa}}}_{i}}^{{\rm{charged}}}\right]$). Then, we can write the following constraints:

30 $$\frac{{\mathrm{d}}}{{\mathrm{d}}t}{T}_{{{\rm{aa}}}_{i}}^{u}=0=-{v}_{{{\rm{aa}}}_{i}}^{{\rm{charging}}}+{\sum _{l\in {\mathcal{L}}}}{\eta }_{{{\rm{aa}}}_{i}}^{l}\cdot {v}_{l}^{{\rm{tsl}}}-\mu * {T}_{{{\rm{aa}}}_{i}}^{u},$$

31 $$\frac{{\mathrm{d}}}{{\mathrm{d}}t}{T}_{{{\rm{aa}}}_{i}}^{c}=0={v}_{{{\rm{aa}}}_{i}}^{{\rm{charging}}}-{\sum _{l\in {\mathcal{L}}}}{\eta }_{{{\rm{aa}}}_{i}}^{l}\cdot {v}_{l}^{{\rm{tsl}}}-\mu * {T}_{{{\rm{aa}}}_{i}}^{c},$$

$$-{v}_{{{\rm{aa}}}_{i}}^{{\rm{charging}}}+\sum _{l\in {\mathcal{L}}}{\eta }_{{{\rm{aa}}}_{i}}^{l}\cdot {v}_{l}^{{\rm{tsl}}}-\mu * {T}_{{{\rm{aa}}}_{i}}^{u}=0,\qquad({{\rm{TB}}}_{{{\rm{aa}}}_{{\rm{i}}}}^{{\rm{u}}})$$

$${v}_{{{\rm{aa}}}_{i}}^{{\rm{charging}}}-\sum _{l\in {\mathcal{L}}}{\eta }_{{{\rm{aa}}}_{i}}^{l}\cdot {v}_{l}^{{\rm{tsl}}}-\mu * {T}_{{{\rm{aa}}}_{i}}^{c}=0.\qquad ({{\rm{TB}}}_{{{\rm{aa}}}_{{\rm{i}}}}^{{\rm{c}}})$$

tRNAs are produced with a charging reaction and consumed by peptide synthesis. We use the following charging reaction:

32 $$\begin{array}{ll}{v}_{{{\rm{aa}}}_{i}}^{{\rm{charging}}}:{{\rm{aa}}}_{i}+{{\rm{tRNA}}}_{{{\rm{aa}}}_{i}}^{{\rm{uncharged}}}+{\rm{ATP}}+2{{\rm{H}}}_{2}{\rm{O}}\hfill\\ \qquad\to {{\rm{tRNA}}}_{{{\rm{aa}}}_{i}}^{{\rm{}}{\mathrm{charged}}}+{\rm{AMP}}+2{{\rm{H}}}^{+}.\end{array}$$

By default, this reaction is assumed to happen spontaneously, but catalytic constraints can be applied if the adequate catalytic rate constants and enzyme compositions are known. Once again, the stoichiometric coefficients of each reactant will appear in the stoichiometric matrix in the column corresponding to this reaction.

### Reformulation of the bilinearity of the problem

The main issue with the EP formulation presented previously lies in the continuous bilinear terms that describe the dilution of the macromolecules, ${G}_{\star }\in \{{E}_{j}\}\cup \left\{{F}_{l}\right\}\cup \{{{\rm{tRNA}}}_{{{\rm{aa}}}_{i}}^{{\rm{(un)charged}}}\}$. We use $\star$ as a placeholder for the indexing of $G$. Using previous notations for the synthesis, degradation, and growth rate:

33 $${v}_{\star }^{{\rm{syn}}}-{v}_{\star }^{{\mathrm{deg}} }-\mu * {G}_{\star }=0.$$

In this state, the dilution term is bilinear, and the formulation requires a bilinear solver or potentially a mixed-integer bilinear solver if thermodynamics are to be added. The original ME-model formulation has similar terms as we are presenting here^6,7^. As such, its recent adaptation in Lloyd et al.^8^ uses the two-level iterative algorithm SolveME^9^ that requires a dedicated nonlinear solver. In this fashion, iterative approaches which try to sequentially improve a value of the growth are a way to deal with the bilinearity. We present instead a MILP approximation of the problem that makes it compatible and solvable with mainstream MILP solvers in a single optimization formulation. We achieve this through the discretization and linearization of the bilinear products. This operation can be understood as locally approximating the bilinear problem by several linear subproblems and choosing the best approximation.

Using a MILP approximation rather than an iterative scheme has two clear advantages. First, it allows to simulate growth-dependent parameters (such as RNA/protein mass ratios) with guarantees on convergence and global optimality directly inherited from the MILP nature of the problem. In the case of parameters that are monotonically increasing or decreasing with respect to growth rate, guarantees exist in quadratically-constrained programming (QCP), such as showed in SteadyCom^38^. However, in the case of non-monotonically increasing or decreasing parameters with respect to the growth rate, such guarantees are harder to prove in general QCP cases, and thus MILP provides a strong framework, with global optimality guarantees and enumeration of alternative solutions. Second, by displacing the solving complexity to the solver, it also allows us to rely on the latest advances in MILP solving, which is a very dynamic field, with new solver releases every 6–12 months.

### Approximation of the growth rate

In ETFL, we approximate the growth rate $\mu$ in bilinear products with a piecewise-constant function $\widehat{\mu }$ (${0}^{{\rm{th}}}$ order approximation). A zeroth-order approximation is an approximation by a piecewise-constant function. If $\widehat{\mu }$ is piecewise-constant, then the product $\widehat{\mu }* {G}_{\star }$ is piecewise-linear. This can be represented in a MILP form, and allows us to transform the continuous bilinear terms into mixed (integer $\times$ continuous) bilinear terms. This simplifies the problem, as these mixed bilinear terms can be linearized in a MILP setting using the Petersen linearization scheme^39^, a particular case of the Glover linearization scheme^40^ that was previously used in metabolic engineering by Hatzimanikatis et al.^41,42^.

Let $\overline{\mu }$ be an upper bound to $\mu$, $\left(p,N\right)\in {N}^{2},p\le N$. We can approximate $\mu$ with the following ${0}^{{\rm{th}}}$ order approximation:

34 $$\forall \mu \in \left[0,\overline{\mu }\right],\qquad \mu \approx \widehat{\mu }=p\cdot \frac{\overline{\mu }}{N}.$$

With this notation, $\frac{\overline{\mu }}{N}$ is, in fact, the resolution of the approximation. $N$ is the number of bins in which $\mu$ has been discretized, and $p$ allows to choose which bin is selected in the solution. For the linearization of the problem, we will need to express $p$ using only binary variables. To this effect, we can perform its binary expansion:

35 $$p={\sum _{s=0}^{\lceil {\mathrm{log}}_{2}N\rceil }}{2}^{s}\cdot {\delta }_{s},$$

where $\lceil {\mathrm{log}}_{2}N\rceil$ denotes the smallest majoring integer to ${\mathrm{log}}_{2}N$, and ${\delta }_{s}\in \{0,1\}$ is ${r}^{{\rm{th}}}$ digits from the right of the binary notation of $p$.

The model needs two more constraint to ensure that $\mu \in \left[\widehat{\mu }-\frac{p}{N},\overline{\mu }+\frac{p}{N}\right]$ and that $p$ does not exceed $N$, which would result in $\widehat{\mu } \, > \, \overline{\mu }$:

36 $$0\le {\sum _{s=0}^{\lceil {\mathrm{log}}_{2}N\rceil }}{2}^{s}\cdot {\delta }_{s}\le N$$

37 $$\quad -\frac{p}{N}\le \mu -\widehat{\mu }\le \frac{p}{N}.$$

As an example, let us consider modeling an organism whose growth rate does not exceed ${\mu }_{{\mathrm{max}}}=2.3\ {{\rm{h}}}^{-1}$. To do this, we can set $\overline{\mu }=2.5\ge {\mu }_{{\mathrm{max}}}$. Let us choose a resolution of $0.25\ {{\rm{h}}}^{-1}$, which gives $N=10$. Then, ${\mathrm{log}}_{2}N\approx 3.32$, and $\lceil {\mathrm{log}}_{2}N\rceil =4$. A growth rate $\mu =1.4$ will be approximated by:

$$	\widehat{\mu } =1.5=6\cdot \frac{\overline{\mu }}{10},\\ 	\widehat{\mu } =\left({\delta }_{0}\times {2}^{0}+{\delta }_{1}\times {2}^{1}+{\delta }_{2}\times {2}^{2}+{\delta }_{3}\times {2}^{3}+{\delta }_{4}\times {2}^{4}\right)\cdot \frac{\overline{\mu }}{10},\\ 	\widehat{\mu } =\left(0\times 1+1\times 2+1\times 4+0\times 8+0\times 16\right)\cdot \frac{\overline{\mu }}{10}.$$

**Fig. 6:**
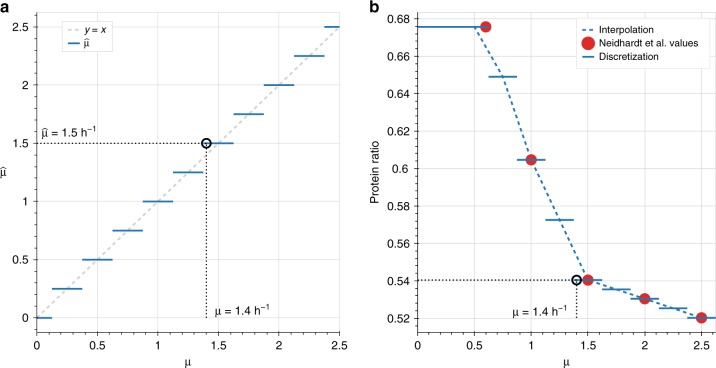
Discretization example for specific growth rate and growth-dependent parameters. **a** Discretization of $\mu$ into $\widehat{\mu }$. The step approximation transforms the continuous interval $\left[0,2.5\right]$ into the discrete set $\left\{0,0.25,\ldots ,2.5\right\}$. **b** Example of piecewise linear interpolation and discretization of the protein mass ratio from Neidhardt et al.^11^. Red circles represent the values reported. The dashed line is the piecewise linear interpolation. The solid line is its discretization.

The values of ${\delta }_{s}$ are obtained by the solver upon optimization. This example is illustrated in Fig. 6a. To maximize the resolution of the model, and minimize the associated computational cost (under the form of three additional constraints for each lineariation to be performed, see Petersen linearization in the Methods section), the user should ideally choose $N$ as a power of two.

MILP solvers use a variety of algorithms and heuristics to solve MILP problems. In this case, the difficulty lies in the fact that the EP and the FBA are almost independent and linked through a limited number of equations and variables. Even though the automated solving methods of the solver might seem obscure to a human, we thought useful to provide a human-understandable heuristic for solving a formulation such as ETFL. It might prove useful in the case where one needs to find an initial non-optimal solution, which sometimes greatly improve solver performances. Thus, conceptually, a heuristic for solving an ETFL problem would be:

1. Solve the FBA for $\mu$
2. Select the corresponding, closest value of $\widehat{\mu }$
3. Apply it to compute dilution values
4. Solve the EP with fixed dilution
5. Apply the catalytic constraints to the FBA
6. Recalculate the FBA under catalytic constraints
7. If $\mu \, \notin \, \big\{\widehat{\mu }\pm \frac{\overline{\mu }}{N}\big\}$, go back to 3, else, end.

### Linearizing the bilinearity

In the previous derivation, we replaced the growth rate variable by a discrete number of acceptable values. We can approximate the continuous product $\mu * {G}_{\star }$, which represents the dilution, as follows:

38 $$\mu * {G}_{\star }\approx \widehat{\mu }* {G}_{\star },$$

39 $$\widehat{\mu }* {G}_{\star }={\sum _{s=0}^{\lceil {\mathrm{log}}_{2}N\rceil }}\frac{{2}^{s}}{N}\overline{\mu}\cdot {\delta }_{s}* {G}_{\star },$$

The product ${\delta }_{l}* {G}_{\star }$ is then still bilinear, but one of its variables is binary. Assuming a constant $M \, > \, {G}_{\star }$, We can use Petersen’s linearization theorem^39,40^ to replace the product ${\delta }_{s}* {G}_{\star }$ with a single nonnegative variable ${z}_{\star }^{s}$, as described in the section Petersen linearization.

Because of the binary expansion, the complexity of the model grows only as ${\mathcal{O}}\left({\mathrm{log}}_{2}N\right)={\mathcal{O}}\left({\mathrm{log}}_{2}\frac{1}{\epsilon }\right)$, where $\epsilon =1/N$ is proportional to the resolution of the approximation (which is $\frac{\overline{\mu }}{N}$). This means that the linearization part of a model with a resolution of $0.01\ {{\rm{h}}}^{-1}$ is only around twofold bigger than that of a model with a resolution $0.04\ {{\rm{h}}}^{-1}$, while resolution has been improved fourfold.

### Petersen linearization

After discretization of the growth rate, the dilution term for the macromolecule ${G}_{\star }$ will consist of a sum of products of the binary variables ${\delta }_{s}$ and the continuous variable ${G}_{\star }$. We can use the Petersen linearization scheme^39^ to transform this product into an equivalent system of one new variable and three new constraints:

$$ {z}_{\star }^{s}={\delta }_{s}* {G}_{\star },\qquad\qquad$$

40 $$\begin{array}{lll}&\iff &\left\{\begin{array}{l}{G}_{\star }+M\cdot {\delta }_{s}-M\le {z}_{\star }^{s}\le M\cdot {\delta }_{s},\\ {z}_{\star }^{s}\le {G}_{\star },\end{array}\right.\\ &\iff &\left\{\begin{array}{l}{G}_{\star }+M\cdot {\delta }_{s}-{z}_{\star }^{s}\le M,\\ {z}_{\star }^{s}-M\cdot {\delta }_{s}\le 0,\,\\ {z}_{\star }^{s}-{G}_{\star }\le 0.\end{array}\right.\end{array}$$

With this method, we can directly reformulate generalized mass balances as described in Eq. (33) for mRNAs, enzymes, uncharged tRNAs, and charged tRNAs:

$${v}_{j}^{{\rm{asm}}}-{v}_{j}^{{\mathrm{deg}} }-\sum _{s=0}^{\lceil {\mathrm{log}}_{2}N\rceil }\frac{{2}^{s}}{N}\overline{\mu }\cdot {z}_{j}^{s}=0,\qquad ({{\rm{EB}}}_{{\rm{j}}}^{{\prime} })$$

$${v}_{l}^{{\rm{tcr}}}-{v}_{l}^{{\mathrm{deg}} }-\sum _{s=0}^{\lceil {\mathrm{log}}_{2}N\rceil }\frac{{2}^{s}}{N}\overline{\mu }\cdot {z}_{l}^{s}=0,\qquad ({{\rm{MB}}}_{{\rm{l}}}^{{\prime} })$$

$$-{v}_{{{\rm{aa}}}_{i}}^{{\rm{charging}}}+\sum _{{{\rm{aa}}}_{i}\in {\mathcal{A}}}{\eta }_{{{\rm{aa}}}_{i}}^{l}\cdot {v}_{l}^{{\rm{tsl}}}-\sum _{s=0}^{\lceil {\mathrm{log}}_{2}N\rceil }\frac{{2}^{s}}{N}\overline{\mu }\cdot {z}_{{{\rm{aa}}}_{i}}^{u,s}=0,\qquad ({{\rm{TB}}}_{{{\rm{aa}}}_{{\rm{i}}}}^{{\prime} {\rm{u}}})$$

$${v}_{{{\rm{aa}}}_{i}}^{{\rm{charging}}}-\sum _{{{\rm{aa}}}_{i}\in {\mathcal{A}}}{\eta }_{{{\rm{aa}}}_{i}}^{l}\cdot {v}_{l}^{{\rm{tsl}}}-\sum _{s=0}^{\lceil {\mathrm{log}}_{2}N\rceil }\frac{{2}^{s}}{N}\overline{\mu }\cdot {z}_{{{\rm{aa}}}_{i}}^{c,s}=0.\qquad ({{\rm{TB}}}_{{{\rm{aa}}}_{{\rm{i}}}}^{{\prime} {\rm{c}}})$$

And we get the additional linearization constraints:

$$\sum _{s=0}^{\lceil {\mathrm{log}}_{2}N\rceil }\frac{{2}^{s}}{N}\overline{\mu }\le \overline{\mu },\qquad (GR)$$

$$-\frac{\overline{\mu }}{2N}\le \mu -\sum _{s=0}^{\lceil {\mathrm{log}}_{2}N\rceil }\frac{{2}^{s}}{N}\overline{\mu }\le \frac{\overline{\mu }}{2N}.\qquad ({\rm{GC}})$$

### Discretization of mRNA and enzyme content

Since the growth has been discretized, it is now possible to also directly discretize other growth-dependent parameters of the problem, regardless of whether they are in a linear or nonlinear relationship with growth. This is a direct consequence of the formulation of ETFL, which allows some flexibility in the modeling assumptions of the user. As an example, we described the relationship between growth and protein and mRNA mass ratios, ${P}^{m}$ and ${R}^{m}$, in the cell as reported in Neidhardt et al.^11^. We thus aim to approximate the nonlinear function ${P}^{m}(\mu )$ (resp. ${R}^{m}(\mu )$) over the interval $\left[0,\overline{\mu }\right]$ with a piecewise-constant function $\widehat{{P}^{m}}$ (resp. $\widehat{{R}^{m}}$). We perform this approximation by interpolating and discretizing the protein ratio and mRNA ratio as functions of the growth rate so that:

41 $$\widehat{{P}^{m}}={\sum _{u\in {\mathcal{U}}}}{\lambda }_{u}\cdot {P}_{u}^{m},$$

42 $$\widehat{{R}^{m}}={\sum _{u\in {\mathcal{U}}}}{\lambda }_{u}\cdot {R}_{u}^{m},$$

where ${P}_{u}^{m}={P}^{m}(u\cdot p\frac{\overline{\mu }}{N})$ (resp. ${R}_{u}^{m}={R}^{m}(u\cdot p\frac{\overline{\mu }}{N})$). ${\lambda }_{u}$ are binary variables, and only one can be active at a time, since we are choosing exactly one value per function. To enforce this behavior, we used a special ordered set constraint of type 1 (SOS1):

$$\sum _{j\in {\mathcal{J}}}{{\rm{MW}}}_{j}\cdot {E}_{j}-\sum _{u\in {\mathcal{U}}}{\lambda }_{u}\cdot {P}_{u}^{m}=0,\qquad ({\rm{IC}}1)$$

$$\sum _{l\in {\mathcal{L}}}{{\rm{MW}}}_{l}\cdot {F}_{l}-\sum _{u\in {\mathcal{U}}}{\lambda }_{u}\cdot {R}_{u}^{m}=0,\qquad ({\rm{IC}}2)$$

$$\sum _{u\in {\mathcal{U}}}{\lambda }_{u}=1.\qquad ({\rm{SOS}}1)$$

${P}_{u}^{m}$ and ${R}_{u}^{m}$ are growth-dependent, interpolated protein and RNA mass ratios (in ${\rm{g}}.{{\rm{g}}}^{-1}$). Given a growth rate, they define the relative mass of the cell that is protein or RNA. $\mathrm{MW}_{\star }$ represents the molar weight of the corresponding enzyme or RNA, and this their product with macromolecules concentrations (in ${\rm{mmol}}.{{\rm{ggDW}}}^{-1}$) will result in mass ratios as well, in grams per gram of dry cell weight. The first two constraints enforce equality between the interpolated data and the model production. The last line is the SOS1 constraint that forces only one of the ${\lambda }_{u}$ to be active.

In addition, it is necessary to have the integer index of ${\lambda }_{u}$ equal to the index of the growth rate. This is obtained through the constraint:

$$\sum _{u\in {\mathcal{U}}}u\cdot {\lambda }_{u}-\sum _{l\in {\mathcal{L}}}{2}^{l}\cdot {\delta }_{l}=0.\qquad ({\rm{EQI}})$$

The first term represents the growth integer index (which discrete value of $\widehat{\mu }$ to use for choosing ${P}_{u}^{m}$), and the second represents its binary expansion (which discrete value of $\widehat{\mu }$ to use for $\mu$). The constraint makes sure they are equal.

Imposing such mass ratios requires the addition of a dummy mRNA as well as a dummy protein to represent the part of the transcriptome/proteome that is either missing from the expression model or altogether unrelated to metabolic function. We use average amino acid frequencies and GC content to model this. Explicit interpolation functions can also be used, such as the growth-dependent functions given by Pramanik et al.^43^.

The simultaneous use of catalytic constraints on metabolic reactions (Eq. ${{\rm{FC}}}_{{\rm{j}}},{{\rm{BC}}}_{{\rm{j}}}$) and maximal enzyme load (Eq. IC3) effectively implements allocation constraints like in GECKO^5^, although in ETFL, the enzyme concentrations are also directly linked to the metabolism. In GECKO, the metabolic cost of building the enzymes is not taken into account.

Figure 6b shows an example piecewise linear interpolation of the growth-dependent protein mass ratio in *E. coli* according to Neidhardt et al.^11^. The reported values (red circles) are interpolated using a piecewise linear function (dashed line), which is then discretized (full line). Using the integer constraints described above, the model can be forced to display a protein content that corresponds to its growth. We apply the same techniques to mRNA and DNA content.

### Discretization of DNA content

To further increase the scope of macromolecules covered by the model, it is also possible to add growth-dependent DNA content. DNA mass ratios at specific growth rates are reported in Neidhardt et al.^11^. We model the DNA reaction synthesis as follows:

$${v}_{{\rm{DNA}}}^{{\rm{synthesis}}}:\, \left(1-\gamma \right){L}_{{\rm{DNA}}}^{{\rm{bp}}}{\rm{dATP}},\\ +\left(1-\gamma \right){L}_{{\rm{DNA}}}^{{\rm{bp}}}{\rm{dTTP}},\\ +\gamma {L}_{{\rm{DNA}}}^{{\rm{bp}}}{\rm{dGTP}},\\ +\gamma {L}_{{\rm{DNA}}}^{{\rm{bp}}}{\rm{dCTP}},\\ \to {\rm{DNA}}+2{L}_{{\rm{DNA}}}^{{\rm{bp}}}{\rm{PPi}},$$

where $\gamma$ is the GC content of the cell, and ${L}_{{\mathrm{DNA}}}^{{\mathrm{bp}}}$ is the total length in base pairs of the DNA. As with ${\mathrm{mRNA}}_{l}$ and ${{\rm{Enz}}}_{j}$, ${\mathrm{DNA}}$ has a mass–balance equation of the following shape:

43 $$\frac{d}{dt}\left[{\rm{DNA}}\right]=0={v}_{{\rm{DNA}}}^{{\rm{synthesis}}}-{v}_{{\rm{DNA}}}^{{\rm{degradation}}}-{v}_{{\rm{DNA}}}^{{\rm{dilution}}},$$

$$\qquad {v}_{{\rm{DNA}}}^{{\rm{synthesis}}}-{v}_{{\rm{DNA}}}^{{\mathrm{deg}} }-\mu * {\rm{DNA}}=0.\qquad ({\mathrm{D}}{{\mathrm{B}}}_{{\mathrm{DNA}}})$$

We consider that the DNA does not degrade, meaning the only source of DNA consumption is dilution caused by the growth of the cell and ${k}_{{\mathrm{deg}} }^{{\rm{DNA}}}=0$. We then define the molar weight of DNA ${{\rm{MW}}}_{{\rm{DNA}}}$ and enforce the DNA mass ratio ${\mathrm{D}}^m$ as we did with both proteins and mRNA:

44 $${{\rm{MW}}}_{{\rm{DNA}}}= 	\;\left(1-\gamma \right){L}_{{\rm{DNA}}}^{{\rm{bp}}}\left({{\rm{MW}}}_{{\rm{dATP}}}+{{\rm{MW}}}_{{\rm{dTTP}}}\right),\\ 	+\,\gamma {L}_{{\rm{DNA}}}^{{\rm{bp}}}\left({{\rm{MW}}}_{{\rm{dGTP}}}+{{\rm{MW}}}_{{\rm{dCTP}}}\right),$$

$${{\rm{MW}}}_{{\rm{DNA}}}\cdot {\rm{DNA}}-\sum _{u\in {\mathcal{U}}}{\lambda }_{u}\cdot {\mathrm{D}}^m_{u}=0.\qquad ({\rm{IC}}3)$$

### Scaling

A critical issue in the formulation of this problem is that the variables are different orders of magnitude. Fluxes are typically between $1{0}^{-3}\!-\!1{0}^{1}\ {\rm{mmol}}.{{\rm{gDW}}}^{-1}.{{\rm{h}}}^{-1}$, whereas protein concentrations are around $1{0}^{-6}-1{0}^{-3}\ {\rm{mmol}}.{{\rm{gDW}}}^{-1}$ and mRNA concentrations are $1{0}^{-10}\!-\!1{0}^{-6}\ {\rm{mmol}}.{{\rm{gDW}}}^{-1}$. The relationship between these scales is given by the catalytic rate constant of enzymes and expression machinery, which spans from $1{0}^{3}\!-\!1{0}^{6}\,{{\mathrm{h}}}^{-1}$. In particular, the ribosome rate constant for translation ($\sim \!12{\rm{aa}}.{{\rm{s}}}^{-1}=43\ 200\ {\rm{aa}}.{{\rm{h}}}^{-1}$) as well as the RNA polymerase rate constant of transcription ($\sim \!85\ {\rm{nt}}.{{\rm{s}}}^{-1}=306\ 000\ {\rm{nt}}.{{\rm{h}}}^{-1}$) are responsible for strong differences in the concentrations and fluxes between transcription- and translation-related parts of the problem. Consequently, the constraint matrix becomes ill-conditioned, and the solver has to operate close to, or sometimes beyond, its maximal solving accuracy (usually around $1{0}^{-9}$ for commercial solvers such as ILOG CPLEX or Gurobi).

To circumvent these limitations, we scale the EP, which will reduce the numerical difficulty of the problem, using nondimensionalization. We create nondimensionalized variables by dividing the variables of the initial problem by an estimated upper bound. For example, by definition, macromolecule concentrations cannot exceed $1{\rm{g}}.{{\rm{gDW}}}^{-1}$, and the following constrains the transformed macromolecule variables between 0 and 1:

45 $$\widehat{X}=\frac{X}{{\sigma }_{X}},{\sigma }_{X}\ge \sup (X)\ \Rightarrow \ 0\le \widehat{X}\le 1.$$

In this scheme, ${\sigma }_{X}$ is an upper bound to $X$. In particular, if we consider ${\sigma }_{X}$ to be the concentration of $1\,{\rm{g}}.{{\rm{gDW}}}^{-1}$:

46 $${\sigma }_{X} 	=1\,{\rm{g}}.{{\rm{gDW}}}^{-1},\\ 	=1\,{\rm{g}}.{{\rm{gDW}}}^{-1}\times \frac{1}{{\rm{MW}}(X)}{\rm{mmol}}.{{\rm{g}}}^{-1},\\ 	=\frac{1}{{\rm{MW}}(X)}{\rm{mmol}}.{{\rm{gDW}}}^{-1},$$

where ${\rm{MW}}(X)$ denotes the molecular weight of the macromolecule in SI units (${\rm{kg}}.{{\rm{mol}}}^{-1}\equiv {\rm{g}}.{{\rm{mmol}}}^{-1}$), and $\widehat{X}$ represents the mass fraction of the molecule in the cell. We scale the fluxes using a method derived from this, detailed in the supporting file Supplementary Note 1. It is also possible to further refine this upper bound by performing a variation analysis on $X$ and re-generating a model using the newly estimated upper bound.

For the sake of clarity, all problem formulations will be kept in their dimensionalized form in the subsequent equations although the implementation is in fact nondimensionalized. The nondimensionalized problem is described further in Supplementary Note 1.

### Advanced modeling

ETFL is amenable to modeling more intricate expression processes. A short selection of these is detailed below.

Enzyme-mediated complex assembly: By default, all the peptides are assumed to assemble spontaneously, without an enzyme. However, in the case of an enzyme-mediated assembly, it is possible to limit the assembly rate by a catalytic constraint if needed, in a fashion similar to Eq. (13). If we denote $A$ the total concentration of assembling enzyme, and ${k}_{\rm{asm}}^{A}$ the catalytic rate constant of assembly, we can constraint ${v}_{j}^{{\rm{asm}}}$ the assembly rate of the ${j}^{{\rm{th}}}$ enzyme:

47 $${v}_{j}^{{\rm{asm}}}\le {k}_{{\rm{asm}}}^{A}\cdot A.$$

Enzyme activation and posttranslational modifications: Some enzymes require to be modified in order to be active, and sometimes by metabolites of the cell. This can be captured by adding a new species representing the active enzyme, and an activation reaction transforming the inactive enzyme to the active form. If the metabolite ${\rm{M}}$ is required to activate enzyme ${{\rm{Enz}}}_{j}$ into ${{\rm{Enz}}}_{j}^{\star }$, then the following activation reaction is added to the model:

48 $${v}_{j}^{\rm{act}}:\qquad {{\rm{Enz}}}_{j}+{\rm{M}}\to {{\rm{Enz}}}_{j}^{\star },$$

The mass balances of ${{\rm{Enz}}}_{j}$ and ${\rm{M}}$ will be supplemented by a term $-{v}_{j}^{{\rm{act}}}$, and the mass balance of ${{\rm{Enz}}}_{j}^{\star }$ by $+{v}_{j}^{{\rm{act}}}$. Finally, the catalytic constraint of the reaction ${v}_{j}$ catalyzed by ${{\rm{Enz}}}_{j}^{\star }$ at concentration ${E}_{j}^{\star }$ shall be:

49 $${v}_{j}\le {k}_{{\rm{cat}}}^{j}\cdot {E}_{j}^{\star }$$

This activation reaction can itself be catalytically limited if needed (see previous paragraph), and require the participation of metabolites. Thus, ETFL allows to capture protein-metabolite interactions.

Enzyme association: It is also possible to model the partition between free enzymes and associated enzymes. In that case, we simply need to operate the following adaptations: (i) replace the ${E}_{j}$ term in any catalytic constraint by a new variable ${E}_{j}^{r}$, which represents the enzymes participating in the catalysis of the ${j}^{{\rm{th}}}$ reaction; (ii) add a variable ${E}_{j}^{F}$ which represents the free enzymes of the system; and (iii) add the enzyme usage constraint:

$${E}_{j}^{r}+{E}_{j}^{F}-{E}_{j}=0\qquad ({{\rm{EU}}}_{{\rm{j}}})$$

Dilution and degradation assumptions: In the current formulation, some species have their dilution or degradation neglected because of high reactivity or slow degradation rate constants. This can be relaxed by simply editing the mass balance reaction according to the assumption to be relaxed. In particular, enzyme-mediated degradation can be modeled by adding suitable catalytic constraints on the degradation reactions. In addition, the dilution term for metabolites can be taken into account if needed, in a manner similar to what Benyamini et al. describe in their method for FBA accounting for dilution^44^.

MILP-based gene knockout strategies for strain design: The ETFL formulation of gene knockout using an upper bound on the translation rate allows to directly formulate MILP-based gene knockout strategies for strain design. Indeed, for each ${l}^{{\rm{th}}}$ gene, we can enforce the constraint:

50 $${v}_{l}^{{\rm{tsl}}}\le M\cdot {b}_{l},$$

with ${v}_{l}^{{\rm{tsl}}}$ the gene’s transcription rate, ${b}_{l}$ a binary variable and $M$ a big-M constant. With that kind of constraint, if $b=1$, the gene is active, while if $b=0$, the gene is knocked-out. It is hence possible to formulate an objective function to optimize the number of KO while fulfilling a metabolic objective, for instance.

### Thermodynamics-based constraints

Thermodynamics flux analysis (TFA)^2,3^ imposes constraints on a FBA problem to couple reaction directionality to the standard free energy of reactions and metabolite concentrations. We also introduce constraints that couple the sign of the Gibbs energy of a reaction to its directionality through the use of integer variables and a mixed-integer linear coupling formulation. This framework reduces the feasible flux space and improves the predictive power of FBA by removing thermodynamically invalid flux profiles.

Considering ${c}_{i}$ is the concentration of ${i}^{{\rm{th}}}$ metabolite, we define ${C}_{i}$ as its scaled logarithm with respect to ${c}_{0}$ so that in standard conditions ${c}_{0}=1\ {\rm{M}}$:

51 $$\forall i,\qquad {C}_{i}={\mathrm{ln}}\left(\frac{{c}_{i}}{{c}_{0}}\right).$$

We use the group contribution method^45^ to directly calculate ${\Delta }_{r}{G}_{j}^{{\prime} \circ }$, the Gibbs energy in solution of the ${j}^{{\rm{th}}}$ reaction. The calculated energy is the net change in the energies of formation of the compounds, which is simply the algebraic sum of the energies of bonds that are broken and formed. This allows to minimize the estimation error of ${\Delta }_{r}{G}_{j}^{{\prime} \circ }$, as there is no error coming from the groups that do not react. Hence, we obtain the additional variables:

52 $${C}_{i}^{\min }\le {C}_{i}\le {C}_{i}^{\max },$$

53 $${\Delta }_{r}{G}_{j,\min }^{{\prime} \circ }\le {\Delta }_{r}{G}_{i}^{{\prime} \circ }\le {\Delta }_{r}{G}_{j,\max }^{{\prime} \circ },$$

54 $${\Delta }_{r}{G}_{j,\min }^{{\prime} }\le {\Delta }_{r}{G}_{i}^{{\prime} }\le {\Delta }_{r}{G}_{j,\max }^{{\prime} }.$$

Some metabolites are not fully characterized, e.g. metabolites with -R groups such as fatty acids, or metabolites attached to a Coenzyme A or acyl-carrier protein. In these cases, the group contribution method allows to directly calculate the net change in the standard Gibbs energy. Since these -R groups are often conserved in the reaction, their contribution terms cancel out when calculating the Gibbs energy of the reaction.

The concentration variables are bounded by experimental measurements or physiological assumptions, and the standard Gibbs energies are bounded by the measurement or estimation error. Since the net flux of each reaction has already been split between forward flux (${v}_{j}^{+}$) and backward flux (${v}_{j}^{-}$), (see Eq. (12)), we can directly add the constraints described in ref. ^2^:

55 $${\Delta }_{r}{G}_{j}^{{\prime} }-{\rm{RT}}{\sum _{i=1}^{m}}{\eta }_{i}^{j}{C}_{i}-{\Delta }_{r}{G}_{i}^{{\prime} \circ }=0,$$

56 $${\Delta }_{r}{G}_{j}^{{\prime} }-K+K\cdot {b}_{j}^{+}\le 0,$$

57 $$-{\Delta }_{r}{G}_{j}^{{\prime} }-K+K\cdot {b}_{j}^{-}\le 0,$$

58 $${v}_{j}^{+}-K\cdot {b}_{j}^{+}\le 0,$$

59 $${v}_{j}^{-}-K\cdot {b}_{j}^{-}\le 0,$$

60 $${b}_{j}^{+}+{b}_{j}^{-}\le 1.$$

${\rm{R}}$ denotes the ideal gas constant, ${\rm{T}}$ is the temperature in Kelvin, and ${\eta }_{i}^{j}$ represents the stoichiometry of the metabolite $i$ in the reaction $j$. $K$ is a big-M constant (bigger than all upper bounds), and ${b}_{j}^{\pm }$ are binary variables. Equation (55) defines the actual Gibbs energy of the reaction as a function of its standard Gibbs energy and the scaled logarithms of metabolite concentrations. Equations (56) and (57) ensure that ${\Delta }_{r}{G}_{j}^{{\prime} }\le 0\iff {b}_{i}^{+}=1$ and ${\Delta }_{r}{G}_{j}^{{\prime} }\ge 0\iff {b}_{i}^{-}=1$. These binary variables are used to block flux in Eqs (58) (59) if the thermodynamics do not favor it. Finally, Eq. (60) is added to enforce that only one direction is chosen.

### Data

mRNA degradation rates constants ${k}_{{\mathrm{deg}} }$ were taken from Bernstein et al.^46^. We converted the reported half lives into rate constants using the classical relationship $k=\frac{\ln(2)}{{t}_{1/2}}$. Proteins were approximated to have a half life of $20\ {\rm{h}}$ (BNID 111930,^47^).

Catalytic rate constants ${k}_{{\rm{cat}}}^{j}$ were obtained from Davidi et al.^48^ for homomeric enzymes. Complex formation reactions for non-homomer enzymes were taken from the supplementary information of O’Brien et al.^7^ and Lloyd et al.^8^. EC numbers were obtained from BiGG^49^ and the iJO1366 publication^12^. Their corresponding ${k}_{{\rm{cat}}}$ values were assigned using conservative (max) values from SabioRK^50^.

Homomer compositions were obtained from Davidi et al.^48^. Other peptide compositions of enzymes were taken from the Supplementary Information of O’Brien et al.^7^ and Lloyd et al.^8^. Additional information was obtained from the Metacyc/Biocyc database^29,51^ using specialized SmartTables queries^52^.

### Model modification

The initial model was subjected to minor changes to accommodate for ETFL modeling. In particular, we added:

- Selenocysteine as a metabolite.
- Cysteine to selenocysteine conversion as a pseudo reaction.
- Replacements for the tRNA metabolites and their charging reaction, as dilution has to be considered.

We also modified the biomass reaction by removing its nucleotide and amino acid components, since they are already taken into account by the expression problem as explained in the section Biomass reaction synthesis and mass balance.

### Enzyme estimation

Given a reaction in the model, if no enzyme is supplied but the reaction possesses a gene reaction rule, it is possible to infer an enzyme from it. The rule expression is expanded, and each term separated by an OR boolean operator is interpreted as an isozyme, while terms separated by an AND boolean operator are interpreted as unit peptide stoichiometric requirements. The enzyme is then assigned an average catalytic rate constant and degradation rate constant.

### Essentiality analysis

The method for testing gene essentiality in FBA is to evaluate for each reaction the gene-protein-reaction association rules (GPRs) containing the gene of interest. The GPR is a boolean expression where the symbols represent whether a gene is expressed. OR operators represent isozymes, and AND operators the assembly of several peptides in a complex. To knock a gene out, its symbol in each GPR is simply assigned the value False. The GPR of all reactions is subsequently evaluated, and the reactions whose GPR evaluates to False are set to have a net flux of 0. Knocking a gene out in ETFL works differently: we replaces GPRs with mass balances, and the direct interaction between gene transcription, peptide translation, enzyme assembly, and metabolism. In this context, knocking-out a gene is done by forcing its transcription rate to 0. Indeed, gene-reactions relationships are conveyed directly through the direct contribution of the relevant peptides either as components of the enzyme complex (AND operator in GPRs) or as isozymes (OR operator). An advantage of this formulation is that it can be used in strain design strategies to optimize directly for knockouts in a single optimization problem.

If a knocked-out gene does not have enzyme associated with it (because of the lack of composition or ${k}_{{\rm{cat}}}$ information), there will be no catalytic constraint associated with the corresponding enzyme. The absence of catalytic constraint will prevent the reaction to be knocked-out. Hence, because of the missing information, gene essentiality information will be lost. An example is the essential reaction Sulfite reductase NADPH2 (SULR). iJO1366 provides a GPR describing a complex needing b2763 and b2764. The ETFL source (the cobraME model and BioCyc) could not provide the stoichiometry of the peptides to form the complex, and thus no enzyme is associated to this reaction in the vETFL model. iJO1366 correctly predicts the genes b2763 and 2764 as essential, but ETFL fails because these genes are not associated to any enzyme. As more enzyme data are added to the model, the false positive rate decreases, as we show in the section Essentiality analysis.

For increased performance, the essentiality analysis was cast into a feasibility problem. We put a lower bound on growth equal to $10 \%$ of the predicted ETFL growth and set the objective to 0. With this method, essential genes will cause the problem to be infeasible, while non-essential genes will return a feasible solution satisfying at least $10 \%$ of the growth. This method achieved up to a fivefold reduction in solving time on the most complex models.

### Hardware

Computations were done on a 64-bit Ubuntu 18.04.1 LTS (Bionic Beaver); 2 $\times$ Intel(R) Xeon(R) CPU E5-2667 v3 @ 3.20 GHz (8 cores, 16 threads per socket); 4 $\times$16 Go @ 2400 MHz RAM. Code was run on Python 3.6 on Docker (18.09.0) containers based on the official python 3.6-stretch container, available on ETFL GitHub and ETFL GitLab.

### Reporting summary

Further information on research design is available in the Nature Research Reporting Summary linked to this article.

## Supplementary information

Supplementary Information

Description of Additional Supplementary Files

Supplementary Data 1

Reporting Summary

## Data Availability

All the data used to conduct this study are available in the organism_data subfolder of the repositories. Some of the data has been obtained from publications, for which all the references are provided in the main text, and a copy has been included in our repositories that mentioned above. The code also contains comments crediting the publications from which data sets and values have been obtained.

## Code availability

The code has been implemented as a plug-in to pyTFA^53^, a Python implementation of the TFA method. It uses COBRApy^54^ and Optlang^55^ as a backend to ensure compatibility with several open source (GLPK, scipy, …) as well as commercial (CPLEX, Gurobi, …) solvers. We rely on the Python package Biopython^56^ for transcribing and translating sequences of nucleotides and amino acids. The code used to generate the models is freely available under the APACHE 2.0 license at https://github.com/EPFL-LCSB/etfl and https://gitlab.com/EPFL-LCSB/etfl.

## References

[1] Magnúsdóttir S (2017). Generation of genome-scale metabolic reconstructions for 773 members of the human gut microbiota. Nat. Biotechnol..

[2] Henry CS, Broadbelt LJ, Hatzimanikatis V (2007). Thermodynamics-based metabolic flux analysis. Biophysical J..

[3] Soh KC, Hatzimanikatis V (2014). Constraining the flux space using thermodynamics and integration of metabolomics data. Methods Mol. Biol..

[4] Beg QK (2007). Intracellular crowding defines the mode and sequence of substrate uptake by escherichia coli and constrains its metabolic activity. Proc. Natl Acad. Sci. USA.

[5] Sanchez BJ (2017). Improving the phenotype predictions of a yeast genome-scale metabolic model by incorporating enzymatic constraints. Mol. Syst. Biol..

[6] Lerman JA (2012). In silico method for modelling metabolism and gene product expression at genome scale. Nat. Commun..

[7] O’Brien EJ, Lerman JA, Chang RL, Hyduke DR, Palsson BO (2013). Genome-scale models of metabolism and gene expression extend and refine growth phenotype prediction. Mol. Syst. Biol..

[8] Lloyd CJ (2018). Cobrame: A computational framework for genome-scale models of metabolism and gene expression. PLoS Computational Biol..

[9] Yang L (2016). solveme: fast and reliable solution of nonlinear me models. BMC Bioinforma..

[10] Ma D (2017). Reliable and efficient solution of genome-scale models of metabolism and macromolecular expression. Sci. Rep..

[11] Neidhardt, F. C. & Curtiss, R. *Escherichia Coli and Salmonella: Cellular and Molecular Biology* Vol. 2 (ASM Press, Washington, DC, 1999).

[12] Orth JD (2011). A comprehensive genome-scale reconstruction of escherichia coli metabolism-2011. Mol. Syst. Biol..

[13] McCloskey D (2014). A model-driven quantitative metabolomics analysis of aerobic and anaerobic metabolism in e. coli k-12 mg1655 that is biochemically and thermodynamically consistent. Biotechnol. Bioeng..

[14] Liebermeister W (2014). Visual account of protein investment in cellular functions. Proc. Natl Acad. Sci. USA.

[15] Otto A (2010). Systems-wide temporal proteomic profiling in glucose-starved bacillus subtilis. Nat. Commun..

[16] Schellenberger J, Palsson BO (2009). Use of randomized sampling for analysis of metabolic networks. J. Biol. Chem..

[17] Schellenberger J (2011). Quantitative prediction of cellular metabolism with constraint-based models: the cobra toolbox v2.0. Nat. Protoc..

[18] Megchelenbrink W, Huynen M, Marchiori E (2014). optgpsampler: an improved tool for uniformly sampling the solution-space of genome-scale metabolic networks. PLoS ONE.

[19] Lee, J., Lam, W. & Dechter, R. Benchmark on daoopt and gurobi with the pascal2 inference challenge problems. (2013). https://www.ics.uci.edu/~dechter/publications/r202.pdf.

[20] Lodi, A. & Tramontani, A. Performance variability in mixed-integer programming. In *Theory Driven by Influential Applications*, 1–12 (INFORMS, 2013). https://pubsonline.informs.org/doi/pdf/10.1287/educ.2013.0112.

[21] CPLEX, I. I. I. High-performance mathematical programming engine. *Int. Business Machines Corp.* (2010). http://www.ibm.com/software/integration/optimization/cplex.

[22] Gu, Z., Rothberg, E. & Bixby, R. *Gurobi Optimizer Reference Manual, Version 8.0*. (Gurobi Optimization Inc., Houston, 2018).

[23] Pandey, V., Hadadi, N. & Hatzimanikatis, V. Enhanced flux prediction by integrating relative expression and relative metabolite abundance into thermodynamically consistent metabolic models. *PLoS Computational Biol.* **15**, e1007036 (2019). 10.1371/journal.pcbi.1007036 PMC6532942 31083653

[24] Zur H, Ruppin E, Shlomi T (2010). imat: an integrative metabolic analysis tool. Bioinformatics.

[25] Becker SA, Palsson BO (2008). Context-specific metabolic networks are consistent with experiments. PLoS Computational Biol..

[26] Pandey, V. & Hatzimanikatis, V. Investigating the deregulation of metabolic tasks via Minimum Network Enrichment Analysis (MiNEA) as applied to nonalcoholic fatty liver disease using mouse and human omics data. *PLoS Computational Biol.* **15**, e1006760 (2019). 10.1371/journal.pcbi.1006760 PMC6493771 31002661

[27] Segre D, Vitkup D, Church GM (2002). Analysis of optimality in natural and perturbed metabolic networks. Proc. Natl Acad. Sci. USA.

[28] Mahadevan R, Edwards JS, Doyle FJ (2002). Dynamic flux balance analysis of diauxic growth in escherichia coli. Biophys. J..

[29] Caspi R (2007). The metacyc database of metabolic pathways and enzymes and the biocyc collection of pathway/genome databases. Nucleic Acids Res..

[30] Arkin AP (2018). Kbase: the united states department of energy systems biology knowledgebase. Nat. Biotechnol..

[31] Fredrickson A (1976). Formulation of structured growth models. Biotechnol. Bioeng..

[32] Milo R, Jorgensen P, Moran U, Weber G, Springer M (2010). Bionumbers-the database of key numbers in molecular and cell biology. Nucleic Acids Res..

[33] Bremer H, Dennis PP (1996). Modulation of chemical composition and other parameters of the cell by growth rate. Escherichia coli Salmonella: Cell. Mol. Biol..

[34] Schuwirth BS (2005). Structures of the bacterial ribosome at 3.5 a resolution. Science.

[35] Zhu J, Penczek PA, Schroder R, Frank J (1997). Three-dimensional reconstruction with contrast transfer function correction from energy-filtered cryoelectron micrographs: procedure and application to the 70s escherichia coli ribosome. J. Struct. Biol..

[36] Gilbert Robert (2009). Physical biology of the cell, by Rob Phillips, Jane Kondev and Julie Theriot. Crystallography Reviews.

[37] Neidhardt, F.C., 1964. The regulation of RNA synthesis in bacteria. In *Progress in nucleic acid research and molecular biology* (Vol. 3, pp. 145–181). Academic Press. 10.1016/s0079-6603(08)60741-2 5318914

[38] Chan SHJ, Simons MN, Maranas CD (2017). Steadycom: predicting microbial abundances while ensuring community stability. PLoS Computational Biol..

[39] Petersen, C. C. *A Note on Transforming the Product of Variables to Linear Form in Linear Programs* (Diskussionspapier, Purdue University, 1971).

[40] Glover F (1975). Improved linear integer programming formulations of nonlinear integer problems. Manag. Sci..

[41] Hatzimanikatis V, Floudas CA, Bailey JE (1996). Analysis and design of metabolic reaction networks via mixed-integer linear optimization. AIChE J..

[42] Hatzimanikatis V, Floudas CA, Bailey JE (1996). Optimization of regulatory architectures in metabolic reaction networks. Biotechnol. Bioeng..

[43] Pramanik J, Keasling J (1997). Stoichiometric model of escherichia coli metabolism: incorporation of growth-rate dependent biomass composition and mechanistic energy requirements. Biotechnol. Bioeng..

[44] Benyamini T, Folger O, Ruppin E, Shlomi T (2010). Flux balance analysis accounting for metabolite dilution. Genome Biol..

[45] Jankowski MD, Henry CS, Broadbelt LJ, Hatzimanikatis V (2008). Group contribution method for thermodynamic analysis of complex metabolic networks. Biophysical J..

[46] Bernstein JA, Khodursky AB, Lin PH, Lin-Chao S, Cohen SN (2002). Global analysis of mrna decay and abundance in escherichia coli at single-gene resolution using two-color fluorescent dna microarrays. Proc. Natl Acad. Sci. USA.

[47] Moran MA (2013). Sizing up metatranscriptomics. ISME J..

[48] Davidi D (2016). Global characterization of in vivo enzyme catalytic rates and their correspondence to in vitro k(cat) measurements. Proc. Natl Acad. Sci. USA.

[49] King ZA (2015). Escher: a web application for building, sharing, and embedding data-rich visualizations of biological pathways. PLoS Computational Biol..

[50] Wittig U., Kania R., Golebiewski M., Rey M., Shi L., Jong L., Algaa E., Weidemann A., Sauer-Danzwith H., Mir S., Krebs O., Bittkowski M., Wetsch E., Rojas I., Muller W. (2011). SABIO-RK--database for biochemical reaction kinetics. Nucleic Acids Research.

[51] Keseler IM (2005). Ecocyc: a comprehensive database resource for escherichia coli. Nucleic Acids Res..

[52] Travers M, Paley SM, Shrager J, Holland TA, Karp PD (2013). Groups: knowledge spreadsheets for symbolic biocomputing. Database.

[53] Salvy, P., Fengos, G., Ataman, M., Pathier, T., Soh, K. C. & Hatzimanikatis, V. pyTFA and matTFA: a Python package and a Matlab toolbox for Thermodynamics-based Flux Analysis. *Bioinformatics* **35**, 167–169 (2018). 10.1093/bioinformatics/bty499 PMC6298055 30561545

[54] Ebrahim A, Lerman JA, Palsson BO, Hyduke DR (2013). Cobrapy: constraints-based reconstruction and analysis for python. BMC Syst. Biol..

[55] Jensen Kristian, G.R. Cardoso Joao, Sonnenschein Nikolaus (2017). Optlang: An algebraic modeling language for mathematical optimization. The Journal of Open Source Software.

[56] Dalke A (2009). Biopython: freely available Python tools for computational molecular biology and bioinformatics. Bioinformatics.

