# Supplementary material for: The ETFL formulation allows multi-omics integration in thermodynamics-compliant metabolism and expression models
Source: Nat Commun. 2020 Jan 13;11:30. doi: 10.1038/s41467-019-13818-7 (PMC6959363; doi:10.1038/s41467-019-13818-7)
Supplement: Supplementary file 1 — Supplementary Information [file 41467_2019_13818_MOESM1_ESM.pdf]

Supplementary Information

The ETFL formulation allows multi-omics integration in thermodynamics-compliant metabolism and expression models

Salvy et al.

Nondimensional Scaling

A critical issue in the formulation of this problem is the different orders of magnitude the variables belong to. Fluxes are typically between $10^{-3} - 10^{1}\ mmol.gDW^{-1}.h^{-1}$. Protein concentrations are around $10^{-6} - 10^{-3}\ mmol.gDW^{-1}$, and mRNA concentrations $10^{-10} - 10^{-6}\ mmol.gDW^{-1}$. The relationship between these scales is given by the catalytic rates of enzymes and expression machinery, which span $10^{3} - 10^{6}\ h^{-1}$. As a consequence, the constraint matrix becomes ill-conditioned and the solver has to operate close to, or sometimes beyond, their maximal solving accuracy (usually around 10^{-9} for commercial solvers such as ILOG CPLEX or Gurobi)

In order to circumvent these limitations, we operate a scaling of the EP which will reduce the numerical difficulty of the problem.

In particular, we consider nondimensionalization by upper bound as a method, which will also allow to reduce the effective range of the variables seen by the solver.

Page 2 details the full bilinear EP formulation, before and after scaling. Page 3 introduces nondimensionalization constants. Page 4 shows the relationship between original variables and scaled variables. Page 5 summarizes upper bounds used for nondimensionalization, and the nondimensionalization variables.

Initial	*Scaled*	
$S \cdot v = 0$		*(FBA)*
Catalytic constraints		
$v_j^f - k_{cat,f}^j E_j \leq 0$	$\tilde{v}_j^f - \tilde{E}_j \leq 0$	*(FC)$_j$*
$v_j^b - k_{cat,b}^j E_j \leq 0$	$\tilde{v}_j^b - \tilde{E}_j \leq 0$	*(BC)$_j$*
Macromolecule mass balances		
$v_j^{asm} - v_j^{deg} - \mu * E_j = 0$	$\tilde{v}_j^{asm} - \tilde{v}_j^{deg} - \frac{\mu}{k_{deg}^j} * \tilde{E}_j = 0$	*(EB)$_j$*
$v_l^{tcr} - v_l^{deg} - \mu * F_l = 0$	$\frac{1}{k_{deg}^l F_l^{ub}} \cdot \frac{k_{cat}^{RNAP} P_l^{ub}}{L_l^{nt}} \cdot \tilde{v}_l^{tcr} - \tilde{v}_l^{deg} - \frac{\mu}{k_{deg}^l} * \tilde{F}_l = 0$	*(MB)$_l$*
$v_l^{tsl} - \sum_j \eta_l^j \cdot v_j^{asm} = 0$	$\tilde{v}_l^{tsl} - \sum_j \eta_l^j \cdot \frac{L_l^{aa}}{k_{cat}^{rib} R_l^{ub}} \cdot k_{deg}^j E_j^{ub} \cdot \tilde{v}_j^{asm} = 0$	*(PB)$_l$*
$-v_{aa_i}^{charging} + \sum_l \eta_{aa_i}^l \cdot v_l^{tsl} - \mu * T_{aa_i}^u = 0$	$-\frac{1}{\bar{\mu}\, T_{aa_i}^{ub}} v_{aa_i}^{charging} + \sum_l \eta_{aa_i}^l \cdot \frac{1}{\bar{\mu}\, T_{aa_i}^{ub}} \cdot \frac{k^{rib} R_l^{ub}}{L_l^{aa}} \cdot \tilde{v}_l^{tsl} - \frac{\mu}{\bar{\mu}} * \tilde{T}_{aa_i}^u = 0$	*(TB)$_{aa,i,u}$*
$v_{aa_i}^{charging} - \sum_l \eta_{aa_i}^l \cdot v_l^{tsl} - \mu * T_{aa_i}^c = 0$	$\frac{1}{\bar{\mu}\, T_{aa_i}^{ub}} v_{aa_i}^{charging} - \sum_l \eta_{aa_i}^l \cdot \frac{1}{\bar{\mu}\, T_{aa_i}^{ub}} \cdot \frac{k^{rib} R_l^{ub}}{L_l^{aa}} \cdot \tilde{v}_l^{tsl} - \frac{\mu}{\bar{\mu}} * \tilde{T}_{aa_i}^u = 0$	*(TB)$_{aa,i,c}$*
$v_{rRNA_l}^{tcr} - v_{rib}^{asm} = 0$	$\frac{1}{k_{deg}^j E_j^{ub}} \cdot \frac{k_{cat}^{RNAP} P_l^{ub}}{L_l^{nt}} \cdot \tilde{v}_{rRNA_l}^{tcr} - \tilde{v}_{rib}^{asm} = 0$	*(RB)$_{rRNA,l}$*
Macromolecule degradation		
$v_j^{deg} - k_{deg}^j \cdot E_j = 0$	$\tilde{v}_j^{deg} - \tilde{E}_j = 0$	*(ED)$_j$*
$v_l^{deg} - k_{deg}^l \cdot F_l = 0$	$\tilde{v}_l^{deg} - \tilde{F}_l = 0$	*(MD)$_l$*
Transcript./translation catalytic constraints		
$v_l^{tsl} - \frac{k_{cat}^{rib}}{L_l^{aa}} R_l \leq 0$	$\tilde{v}_l^{tsl} - \tilde{R}_l \leq 0$	*(TR2)$_l$*
$v_l^{tcr} - \frac{k_{cat}^{RNAP}}{L_l^{nt}} P_l \leq 0$	$\tilde{v}_l^{tcr} - \tilde{P}_l \leq 0$	*(TR1)$_l$*
Expression coupling		
$R_l - \frac{L_l^{nt}}{L_{rib}^{nt}} F_l \leq 0$	$\tilde{R}_l - \frac{L_l^{nt}}{L_{rib}^{nt}} \frac{F_l^{ub}}{R_l^{ub}} \tilde{F}_l \leq 0$	*(EX)$_l$*
Capacity constraints		
$R_F - (1-\rho) E_{rib} = 0$	$\tilde{R}_F - (1-\rho) \tilde{E}_{rib} = 0$	*(RR)*
$\sum_l R_l + R_F - E_{rib} = 0$	$\sum_l \frac{R_l^{ub}}{E_{rib}^{ub}} \tilde{R}_l + \tilde{R}_F - \tilde{E}_{rib} = 0$	*(TC2)*
$\sum_l P_l - E_{RNAP} = 0$	$\sum_l \frac{P_l^{ub}}{E_{RNAP}^{ub}} P_l - \tilde{E}_{RNAP} = 0$	*(TC1)*

Initial	***Scaled***	
$S \cdot v = 0$		*(FBA)*
Catalytic constraints		
$v_j^f - k_{cat,f}^j E_j \leq 0$	$\tilde{v}_j^f - \tilde{E}_j \leq 0$	*(FC)$_j$*
$v_j^b - k_{cat,b}^j E_j \leq 0$	$\tilde{v}_j^b - \tilde{E}_j \leq 0$	*(BC)$_j$*
Macromolecule mass balances		
$v_j^{asm} - v_j^{deg} - \mu * E_j = 0$	$\tilde{v}_j^{asm} - \tilde{v}_j^{deg} - \alpha_j \cdot \mu * \tilde{E}_j = 0$	*(EB)$_j$*
$v_l^{tcr} - v_l^{deg} - \mu * F_l = 0$	$\gamma_l \cdot \tilde{v}_l^{tcr} - \tilde{v}_l^{deg} - \beta_j \cdot \mu * \tilde{F}_l = 0$	*(MB)$_l$*
$v_l^{tsl} - \sum_j \eta_l^j \cdot v_j^{asm} = 0$	$\tilde{v}_l^{tsl} - \sum_j \eta_l^j \cdot \delta_l^j \cdot \tilde{v}_j^{asm} = 0$	*(PB)$_l$*
$-v_{aa_i}^{charging} + \sum_l \eta_{aa_i}^l \cdot v_l^{tsl} - \mu * T_{aa_i}^u = 0$	$-\frac{1}{\bar{\mu}\, T_{aa_i}^{ub}} v_{aa_i}^{charging} + \sum_l \eta_{aa_i}^l \cdot \tau_l \cdot \tilde{v}_l^{tsl} - \frac{\mu}{\bar{\mu}} * \tilde{T}_{aa_i}^u = 0$	*(TB)$_{aa,i,u}$*
$v_{aa_i}^{charging} - \sum_l \eta_{aa_i}^l \cdot v_l^{tsl} - \mu * T_{aa_i}^c = 0$	$\frac{1}{\bar{\mu}\, T_{aa_i}^{ub}} v_{aa_i}^{charging} - \sum_l \eta_{aa_i}^l \cdot \tau_l \cdot \tilde{v}_l^{tsl} - \frac{\mu}{\bar{\mu}} * \tilde{T}_{aa_i}^u = 0$	*(TB)$_{aa,i,c}$*
$v_{rRNA_l}^{tcr} - v_{rib}^{asm} = 0$	$\gamma_l \cdot \tilde{v}_{rRNA_l}^{tcr} - \tilde{v}_{rib}^{asm} = 0$	*(RB)$_{rRNA,l}$*
Macromolecule degradation		
$v_j^{deg} - k_{deg}^j \cdot E_j = 0$	$\tilde{v}_j^{deg} - \tilde{E}_j = 0$	*(ED)$_j$*
$v_l^{deg} - k_{deg}^l \cdot F_l = 0$	$\tilde{v}_l^{deg} - \tilde{F}_l = 0$	*(MD)$_l$*
Transcript./translation catalytic constraints		
$v_l^{tsl} - \frac{k_{cat}^{rib}}{L_l^{aa}} R_l \leq 0$	$\tilde{v}_l^{tsl} - \tilde{R}_l \leq 0$	*(TR2)$_l$*
$v_l^{tcr} - \frac{k_{cat}^{RNAP}}{L_l^{nt}} P_l \leq 0$	$\tilde{v}_l^{tcr} - \tilde{P}_l \leq 0$	*(TR1)$_l$*
Expression coupling		
$R_l - \frac{L_l^{nt}}{L_{rib}^{nt}} F_l \leq 0$	$\tilde{R}_l - \lambda_l \cdot \tilde{F}_l \leq 0$	*(EX)$_l$*
Capacity constraints		
$R_F - (1-\rho) E_{rib} = 0$	$\tilde{R}_F - (1-\rho)\tilde{E}_{rib} = 0$	*(RR)*
$\sum_l R_l + R_F - E_{rib} = 0$	$\sum_l \rho_l \cdot \tilde{R}_l + \tilde{R}_F - \tilde{E}_{rib} = 0$	*(TC2)*
$\sum_l P_l - E_{RNAP} = 0$	$\sum_l \pi_l \cdot P_l - \tilde{E}_{RNAP} = 0$	*(TC1)*

Variable	*Nondimensionalization factor*	*Scaled variable*
X	α	$\tilde{X} = \frac{X}{\alpha}$
E_j	E_j^{ub}	$\tilde{E}_j = \frac{E_j}{E_j^{ub}}$
F_l	F_l^{ub}	$\tilde{F}_l = \frac{F_l}{F_l^{ub}}$
R_l	R_l^{ub}	$\tilde{R}_l = \frac{R_l}{R_l^{ub}}$
R_F	E_{rib}^{ub}	$\tilde{R}_F = \frac{R_F}{E_{rib}^{ub}}$
P_l	P_l^{ub}	$\tilde{P}_l = \frac{P_l}{P_l^{ub}}$
$T_{aa_i}^u$	$T_{aa_i}^{ub}$	$\tilde{T}_{aa_i}^u = \frac{T_{aa_i}^u}{T_{aa_i}^{ub}}$
$T_{aa_i}^c$	$T_{aa_i}^{ub}$	$\tilde{T}_{aa_i}^c = \frac{T_{aa_i}^c}{T_{aa_i}^{ub}}$
v_j^f	$k_{cat,f}^j E_j^{ub}$	$\tilde{v}_j^f = \frac{v_j^f}{k_{cat,f}^j E_j^{ub}}$
v_j^b	$k_{cat,b}^j E_j^{ub}$	$\tilde{v}_j^b = \frac{v_j^b}{k_{cat,b}^j E_j^{ub}}$
v_j^{deg}	$k_{deg}^j E_j^{ub}$	$\tilde{v}_j^{deg} = \frac{v_j^{deg}}{k_{deg}^j E_j^{ub}}$
v_l^{deg}	$k_{deg}^l F_l^{ub}$	$\tilde{v}_l^{deg} = \frac{v_l^{deg}}{k_{deg}^l F_l^{ub}}$
v_l^{tsl}	$\frac{k^{rib}}{L_l^{aa}} R_l^{ub}$	$\tilde{v}_l^{tsl} = \frac{L_l^{aa}}{k_{cat}^{rib} R_l^{ub}} \cdot v_l^{tsl}$
v_l^{tcr}	$\frac{k_{cat}^{RNAP}}{L_l^{nt}} P_l^{ub}$	$\tilde{v}_l^{tcr} = \frac{L_l^{nt}}{k_{cat}^{RNAP} P_l^{ub}} \cdot v_l^{tcr}$
v_j^{asm}	$k_{deg}^j E_j^{ub}$	$\tilde{v}_j^{asm} = \frac{v_j^{asm}}{k_{deg}^j E_j^{ub}}$

Upper bound	***(Approximate) Value***	***Order of magnitude***	***Unit***
E_j^{ub}	$\frac{1}{MW\left(Enz_j\right)} \cdot 10^3$	$10^{-2} \cdot 10^3 = 10^1$	$\frac{[g.gDW^{-1}]}{[g.mol^{-1}]}\left[\frac{mmol}{mol}\right] = mmol.gDW^{-1}$
F_l^{ub}	$\frac{1}{MW(mRNA_l)} \cdot 10^3$	$10^{-2} \cdot 10^3 = 10^1$	$mmol.gDW^{-1}$
R_l^{ub}	$\frac{1}{MW(Rib)} \cdot 10^3$	$10^{-6} \cdot 10^3 = 10^{-3}$	$mmol.gDW^{-1}$
P_l^{ub}	$\frac{1}{MW(RNAP)} \cdot 10^3$	$10^{-5} \cdot 10^3 = 10^{-2}$	$mmol.gDW^{-1}$
$T_{aa_i}^{ub}$	$\frac{1}{MW\left(tRNA_{aa_i}\right)} \cdot 10^3$	$10^{-2} \cdot 10^3 = 10^1$	$mmol.gDW^{-1}$

Nondimensionalization term	***Expression***
α_j	$\frac{\mu}{k_{deg}^j}$
β_l	$\frac{\mu}{k_{deg}^l}$
γ_l	$\frac{1}{k_{deg}^l F_l^{ub}} \cdot \frac{k_{cat}^{RNAP} P_l^{ub}}{L_l^{nt}}$
δ_l^j	$\frac{L_l^{aa}}{k_{cat}^{rib} R_l^{ub}} \cdot k_{deg}^j E_j^{ub}$
$\tau_l^{aa_i}$	$\frac{1}{\bar{\mu}\, T_{aa_i}^{ub}} \cdot \frac{k^{rib} R_l^{ub}}{L_l^{aa}}$
λ_l	$\frac{L_l^{nt}}{L_{rib}^{nt}} \frac{F_l^{ub}}{R_l^{ub}}$
ρ_l	$\frac{R_l^{ub}}{E_{rib}^{ub}}$
π_l	$\frac{P_l^{ub}}{E_{RNAP}^{ub}}$

Supplementary Table 1: Example EP constraint matrix.

	FBA fluxes	Biomass reaction flux	tRNA charging reactions	Translation fluxes	Transcription fluxes	Degradation fluxes	Complexation fluxes	Enzyme Concentrations	Ribosome concentrations	RNAP Concentrations	tRNA Concentrations	mRNA Concentrations	DNA concentration	Growth discretization variables	Product variables	Allocation indicator variables	Allocation variables	**61301**
FBA mass balances	■	■	■	■	■	■												1806
Peptide mass balances				■			■											1431
rRNA mass balances					■		■											3
Catalytic constraint	■							■										862
Translation				■					■									1431
Translation capacity									■									2
Transcription					■					■								1431
Transcription capacity										■								2
Transcription-translation coupling									■			■						1430
Enzyme mass balances						■	■		■	■					■			562
tRNA mass balances			■	■											■			42
mRNA mass balances					■	■									■			1431
DNA mass balance													■				■	1
Degradation definition						■		■	■	■	■	■						1993
Product linearization								■	■	■	■	■		■	■			48864
Growth coupling		■												■				1
Growth discretization														■				1
AllocationConstraints								■	■	■		■	■	■		■	■	8
**37500**	5168	2	42	2862	2862	3986	1124	562	1431	1431	42	1431	1	9	16288	256	3	

Standard Operating Procedure to construct an ETFL model.

Summary checklist

Here is a summarized checklist of the material needed to turn a COBRA model into ETFL:

- A working installation of ETFL
- A Cobra model with:
  - Gene identifiers (IDs)
  - All nucleotides triphosphates(NTPs), deoxynucleotides triphosphate(dNTP), nucleotides monophosphate (NMP), aminoacids.
  - (Optional) Gene reaction rules
- Gene sequences indexed by their gene IDs
- Peptide stoichiometry of enzymes
- Enzyme assigments per reaction.
- Enzyme catalytic rate constants:
  - Forward
  - (Optional) Reverse
- Enzyme degradation rate constants
- mRNA degradation rate constants
- (Optional) Free ribosomes ratio
- (Optional) Free RNA Polymerase ratio
- (Optional) GC-content and length of the genome
- (Optional) Average aminoacid abundances
- (Optional) Average NTP abundances
- (Optional) Average mRNA length
- (Optional) Average peptide length
- (Optional) Growth-dependant mRNA, peptide, and DNA mass ratios.

Setup

Prerequisites

Make sure you have `Git` installed. Since ETFL is built upon pyTFA [1], we will clone both repositories. In a folder of your choice, download the source code from our repositories:

```
git clone https://github.com/EPFL-LCSB/pytfa
git clone https://github.com/EPFL-LCSB/etfl
# -- OR --
git clone https://gitlab.com/EPFL-LCSB/pytfa
git clone https://gitlab.com/EPFL-LCSB/etfl
```

Docker container (recommended)

We recommend the use of Docker containers as they provide a standardized, controlled and reproducible environment. The ETFL Docker is built upon the pyTFA Docker image. We recommend building it yourself as it is where your solvers can be installed.

Downloading Docker

If Docker is not yet installed on your machine, you can get it from [here]

Building and running the Docker container

```
# Build the pyTFA docker
cd pytfa/docker && . build
# Build and run the ETFL docker
cd ../../etfl/docker
. build
. run
```

Solvers

For installing the solvers, please refer to the pyTFA documentation

Python environment

Alternatively, you can install ETFL using `pip`:

```
pip install etfl
```

Make sure your solvers are also installed in the same environment if you are using a `virtualenv` or `pyenv`.

From COBRA to ETFL

ETFL models can be generated fairly easily from a COBRA model. In the following subsections, we detail the required information to add expression constraints to a COBRA model and turn it into an ETFL model.

Constraint-based model

You will need to start with a COBRA model including the following information:

- Genes and their gene ID (necessary to retrieve gene sequences)
- (Optional) Gene-protein rules: These are used to make approximated enzymes if peptide information is not enough

Additionally, you will need to build a dictionnary of essential metabolites required in the model. It should follow this example structure (all fields mandatory):

```
dict(atp='atp_c',
     adp='adp_c',
     amp='amp_c',
     gtp='gtp_c',
     gdp='gdp_c',
     pi ='pi_c' ,
     ppi='ppi_c',
     h2o='h2o_c',
     h  ='h_c'  )
```

A dictionnary of RNA NTPs, DNA dNTPS, and aminoacids is also required, of the type:

```
aa_dict = {'A': 'ala__L_c',
           # ...
           'V': 'val__L_c', }

rna_nucleotides = {
                'u': 'utp_c',
                # ...
                'c': 'ctp_c'}

rna_nucleotides_mp = {
                'u': 'ump_c',
                # ...
                'c': 'cmp_c'}

dna_nucleotides = {
                't': 'dttp_c',
                # ...
                'c': 'dctp_c'}
```

From genes to peptides

In order to build the transcription and translation, it is necessary to provide ETFL with gene deoxynucleotide sequences. These will be automatically transcribed in RNA sequences and then translated into aminoacid peptide sequences. They must be fed to the function `model.add_nucleotides_sequences` in a

dict-like object, indexed by gene IDs (`model.genes.mygene.id` property in COBRA).

We suggest the following sources for obtaining such information:

- KEGG Genes
- NCBI Gene DB
- MetaCyc Gene Search

ETFL will automatically synthesize the correct peptides from the nucleotides sequences. This is based on the Biopython package's `transcribe` and `translate` functions [2].

For each enzyme created by transcription, a degradation rate constant must be specified. These can be obtained through literature search, or using an average value.

From peptides to enzymes

A key part of the expression modeling is to properly represent the assembly of enzymes from peptides. For each enzyme of the model, a stoichiometry of the peptides necessary for its assembly is needed. These are stored as dictionnaries in the `Enzyme.composition` property under a form similar to :

```
>>> enzyme.composition
{'b2868': 1, 'b2866': 1, 'b2867': 1}
```

The keys match the IDs of genes coding for the peptide, and the value represent the stoichiometry of the peptide in the enzyme. These can be obtained from litterature search or specialized databases. In particular, we used for this paper the Metacyc/Biocyc database [3, 4], using specialised SmartTables queries [5].

```
html-sort-ascending(
    html-table-headers (
        [(f,genes,(protein-to-components f)):
            f<-ECOLI^^Protein-Complexes,genes := (enzyme-to-genes f)
        ],
        ("Product Name", "Genes", "Component coefficients")),
    1)
```

At this step, it is also possible to implement post-translational changes or enzyme-specific mechanisms. The assembly reaction of peptides can be edited like any normal reaction to include other metabolites, for example metal ions.

From enzymes back to the metabolism

Lastly, the enzymes must be assigned reactions and catalytic rate constants. Several enzymes can catalyze the same reactions. COBRA models can take this into account differently, usually having either (i) multiple reactions with a simple gene reaction rule; or (ii) one unique reaction with several isozymes in the gene reaction rule. Although not often applied consistently within the same

model, these two formalisms are equivalent, and their ETFL counterparts will also behave equivalently.

For each enzyme, the information needed is the (forward) catalytic rate constant k^+_{cat}, facultatively the reverse catalytic rate constant k^-_{cat} (set equal to k^+_{cat} if none is given), and a degradation rate constant.

This is done by calling the function `model.add_enzymatic_coupling(coupling_dict)` where `coupling_dict` is a dict-like object with reaction IDs as keys and a list of enxyme objects as values:

```
coupling_dict = {
    #...
    'AB6PGH': [ <Enzyme AB6PGH_G495_MONOMER at 0x7ff00e0f1b38>],
    'ABTA'  : [ <Enzyme ABTA_GABATRANSAM at 0x7ff00e0fda90>,
                <Enzyme ABTA_G6646 at 0x7ff00e0fd4e0>],
    'ACALD' : [ <Enzyme ACALD_MHPF at 0x7ff00e0fdcf8>],
    #...
    }
```

The catalytic rate constants can be obtained from several databases, such as:

- Rhea
- BRENDA
- SabioRK
- Uniprot

Several enzymes can be assigned to a reaction. ETFL will try to match the gene reaction rule isozymes to the supplied enzymes. If the gene reaction rule shows several isozymes while only one enzyme is supplied, the enzyme can be replicated to match the number of isozymes in the gene reaction rule.

Given a reaction in the model, if no enzyme is supplied but the reaction possesses a gene reaction rule, it is possible to infer an enzyme from it. The rule expression is expanded, and each term seprated a by an `OR` boolean operator is interpreted as an isozyme, while terms separated by an `AND` boolean operators are interpreted as unit peptide stoichiometric requirements. The enzyme is then assigned an average catalytic rate constant and degradation rate constant.

Growth-dependant parameters

Accounting for growth-dependent RNA and protein content requires additional information. In particular:

- GC-content and length of the genome
- Average aminoacid abundances
- Average NTP abundances
- Average mRNA length

- Average peptide length
- Growth-dependant mRNA, peptide, and DNA mass ratios.

These values are usually obtained through litterature search. All of the last three ratios are optional, although using none defeats the purpose of accounting for growth-dependant parameters.

Additional documentation

Example

We encourage the reader to look at the script used to generate the models with which the paper's results were generated, available in `etfl/tutorials/helper_gen_models.py`. The data it takes in input has been generated in `etfl/etfl/data/ecoli.py`. These are good examples to start from in order to make a custom ETFL from a different COBRA model.

Note on steady-state assumptions and dilution terms.

Flux balance analysis (FBA) is an important tool in metabolic engineering to analyze the stoichiometric properties of living systems. Its success is partly due to the simplicity of its formulation as a linear program, with a constraint matrix of the form $S \cdot v = 0$, where S is the stoichiometric matrix of the system of interest, and v the biochemical fluxes carried by the reactions in the system. This formulation is directly derived from the mass balance of the metabolites inside the cell. The simplicity of this formulation stems from two important assumptions: (i) in the mass balance of the metabolites, the dilution term is negligible ; and (ii) the intracellular concentrations of the metabolites are at quasi-steady state.

The main purpose of ME-models is to account for macromolecule synthesis costs on top of a metabolic model. However, the macromolecular concentrations are subject to different assumptions. In particular, when writing the mass balances for the said macromolecules, the dilution term is not negligible anymore. Furthermore, the quasi-steady state assumption applies on a different timescale, since macromolecules synthesis rates are several orders of magnitude slower than metabolic reactions.

Here we present three arguments to explain and justify the assumptions made in ETFL and the form of the mass balance equations for metabolites and macromolecules. We briefly discuss these arguments for the case of metabolites and contrast them in the case of macromolecules, to study the validity of the assumptions made in ETFL.

Preliminaries

The mass balances of biochemical species is written with respect to their concentration variables. If we assume the cell is growing at a specific growth rate μ, we must assume that the volume of cell within which the mass balance is considered varies.

The mass balance of a compound X can be expressed both as the derivative of the mass or the algebraic sum of its synthesis and consumption fluxes:

$$\frac{dm_X}{dt} = C_X \frac{dV_c}{dt} + V_c \frac{dC_X}{dt} \tag{1}$$

$$= S_X^\top \cdot v \cdot V_c, \tag{2}$$

where C_X is the concentration of compound X in the cellular volume V_c, for a total mass m_X in the cell, and whose stoichiometry with respect to the fluxes v is described by the row S_X of the stoichiometric matrix S.

We next combine equations 1 and 2 and divide by V_c (necessarily non-zero) to write the time derivative of the concentration C_X:

$$\frac{dC_X}{dt} = S_X^\top \cdot v - \frac{1}{V_c} \frac{dV_c}{dt} \cdot C_X. \tag{3}$$

By definition, $\frac{1}{V_c}\frac{dV_c}{dt} = \mu$ is the specific growth rate of the cell (under the assumption of constant cell density ρ_c), and the term $\mu \cdot C_X$ is called the dilution term, as per Fredrickson's work on formulating growth models [6]. We can hence write the general mass balance of a biochemical species in the cell as:

$$\frac{dC_X}{dt} = S_X^\top \cdot v - \mu \cdot C_X. \tag{4}$$

In this equation, rates are in g/(L · h), and concentrations in g/L. If we divide rates and concentrations by their respective molecular weight and the mass of one liter of dried cells, their units become respectively mmol/(gDW · h), and mmol/gDW. We will use the latter unit system in the rest of this note.

Intracellular fluxes and dilution

In FBA, the dilution term is omitted from the mass balance of the metabolites. In ETFL, this term is also omitted for metabolites, but preserved for macromolecules. We present here two arguments which support the fact that the contribution of the dilution is negligible for metabolites, but not for macromolecules.

Orders of magnitude argument

The average metabolite concentration in the cells do not exceed 10^{-2} M = 10 mmol/L [7]. Assuming the cell has a density close to 1 kg/L = 1000 g/L, and that $0.5 \approx 10^0$ gDW/g of dry cells is obtained per gram of culture, we derive that:

Intracellular metabolite concentrations are upperbounded by 10^{-2} mmol/gDW.

From typical FBA results and flux variability analyses, we can claim the following:

Metabolic fluxes typically range from 10^{-2} to 10^1 mmol/(gDW · h).

These fluxes are higher close to the carbon uptake, in the central carbon metabolism, and decrease in the more distant pathways.

One *E. coli* cell weighs 1 pg. Using the previous constants yields a conversion factor of 10^{-8} (mmol$_{\text{cell}}$)/gDW). The number of mRNA copies per cell per transcript is in the order of magnitude 10^0 copies/cell (BNID 112795 [8]). This amounts to a typical mRNA concentration of 10^{-8} mmol$_{\text{mRNA}}$/gDW. Protein-to-mRNA ratios are typically ranging from 10^2 to 10^4 proteins/mRNA (BNID 106254 [9]).

From this we can assert:

Intracellular macromolecule concentrations range from 10^{-8} to 10^{-4} mmol/gDW.

There are in average 6.6 ribosomes per thousand base pairs per cell (BNID 107727 [10]), and the average transcript is around 1 kb, with one copy per transcript, which amounts to 10^1 mmol$_{\text{rib}}$/(cell · transcript). The translation rate per ribosome is 10 aa/(s · ribosome) (BioNumbers ID [BNID] 100059 [11]) This gives an upper bound on the specific peptide synthesis fluxes of $v^{tsl} \approx$

10^{-6} mmol/(gDW · h) Using the protein-to-mRNA ratio allows us to estimate an upperbound on transcription rates from 10^{-10} to 10^{-8} mmol/(gDW · h).

This yields:

> The typical macromolecule synthesis rate range from 10^{-10} to 10^{-6} mmol/(gDW · h).

An interesting intermediary case to consider is that of macromolecule monomers (nucleotides for mRNA and amino acids for peptides). Under the assumption that the typical protein is ≈ 325 amino acids long (BNID 108986 [12]), the average mRNA transcript is ≈ $1kb$, and there are ≈ 10^3 different mRNAs and peptides, we can derive typical monomer concentrations for each of the ≈ 20 amino acids and 4 nucleotides. Thus, nucleotides have a typical concentration of 10^{-3} mmol_{nt}/gDW, and amino acids have a typical concentration of 10^{-3} to 10^{-1} mmol_{aa}/gDW. We can then assert:

> Macromolecule monomers have a typical concentration of 10^{-3} to 10^{-1} mmol/gDW.

Assuming that 50% of the glucose goes towards protein synthesis, and that all the ≈ 20 amino acids are synthesized in similar amounts at yields between 0.5 and 2.0 $\text{mol}_{\text{aa}}/\text{mol}_{\text{glc}}$ [13], the amino acid biosynthesis fluxes are one to two orders of magnitude smaller than those of the central carbon metabolism. Nucleotide synthesis is even smaller. From there, we can claim:

> Monomer synthesis fluxes range from 10^{-2} to 10^{-1} mmol/(gDW · h)

The values we obtain for the elements of Eq. 4 are detailed in Table 3. The table shows clearly that, in the case of metabolites, the dilution term is negligible in front of the metabolic fluxes. It also shows that for macromolecule monomers, which are further away from the central carbon metabolism, the dilution term becomes comparable with the synthesis term. These orders of magnitude are in agreement with the comprehensive discussion on the magnitude of pools, metabolic fluxes, and dilution terms for different metabolites in the cell (including central carbon pathway and amino acids) featured in the chapter 8.1 of the work by Stephanopoulos, Aristidou and Nielsen [14]. Finally, the range of synthesis fluxes for macromolecules greatly overlaps with that of their dilution term, which imposes taking the dilution into account.

Yield argument

We have seen before that the average amino acid yield per molecule of glucose metabolised is between 0.5 and 2.0 $\text{mol}_{\text{aa}}/\text{mol}_{\text{glc}}$ [13]. The average protein is

Supplementary Table 2: Orders of magnitudes of the variables in presence for the mass balance of metabolites.

Variable	Order of magnitude			Units
	Metabolites	Monomers	Macromolecules	
$S_X^\top \cdot v$	$10^{-2} - 10^{1}$	$10^{-2} - 10^{-1}$	$10^{-10} - 10^{-6}$	mmol/(gDW · h)
$\mu \cdot C_X$	$10^{-3} - 10^{-2}$	$10^{-4} - 10^{-1}$	$10^{-9} - 10^{-4}$	mmol/(gDW · h)

made of $\approx$ 325 amino acids (BNID 108986 [12]). This implies that the global protein synthesis rate is at least 2 orders of magnitude slower than those of the central carbon metabolism. Given approximately 10^3 different peptides, one can expect specific peptide synthesis rates to be at their maximum 5 orders of magnitude smaller than central carbon metabolism fluxes.

This estimation matches with the results presented in Table 3.

An additional note about accounting for dilution in FBA

Benyamini *et al.* [15] report on the results of FBA accounting for dilution terms, a method called MD-FBA. Their results show a sensitivity in gene essentiality analysis, especially for genes far from the central carbon pathways, and closer to the biomass precursor pathways. In particular, the fluxes in which the dilution term has a significant impact are the fluxes close to the synthesis of the macromolecule monomers.

This can be understood in the context of the order-of-magnitude argument since breaking up the glucose to piece together biomass precursors further splits the available carbon between the different precursors, thus reducing their synthesis fluxes, which then become comparable to the dilution rate.

Taken to the extreme, this reasoning matches the yield argument made previously, where specific peptide synthesis fluxes will be several orders of magnitude smaller that the central metabolism fluxes, making the dilution term non-negligible.

Timescale analysis

Heineken *et al.*'s analysis of the pseudo-steady state hypothesis for biochemical kinetics [16] provides a method to study kinetic equations using non-dimensionalization. We adapt this method to Eq. 4 to derive a justification of the pseudo-steady state hypothesis.

Let us assume that the general flux term $v(t)$ can be written as the product of a diagonal matrix of catalytic rate constants K and a function of concentration of the compounds taking part in the reactions $\Phi(C,t)$, with $C(t) = (C_X(t))_{X\in\text{species}}$. This product can represent, for instance, either mass action kinetics, or Michaelis-Menten kinetics. We can hence rewrite Eq. 4:

$$\frac{dC_X(t)}{dt} = S_X^\top \cdot K \cdot \Phi(C,t) - \mu \cdot C_X(t). \tag{5}$$

In a Michaelis-Menten case, for example, the elements of the matrix K will represent the catalytic rate constants k_{cat}.

Quasi-steady state assumption

We introduce the dimensionless variables:

$$\tau = kt, \qquad z(\tau) = \frac{C_X(\tau/k)}{c_0}, \qquad y(\tau) = \frac{\Phi(C,\tau/k)}{c_0}, \tag{6}$$

where k is an inverse time constant of our choice and c_0 is the average species concentration, acting as a non-dimensionalization factor. We can rewrite Eq. 5:

$$\frac{dz}{d\tau} = S_X^\top \cdot \left(\frac{1}{k}K\right) \cdot y(\tau) - \frac{\mu}{k} \cdot z(\tau) = F(y, z, \tau). \quad (7)$$

In Heineken's words, if k is sufficiently large, τ *represents time vastly accelerated* so that $F(y, z, \tau)$ is held at a stable root of $F(y, z, \tau) = 0$ (which exists if $\Phi(C, t)$ is sufficiently well-behaved, by following for instance Michaelis-Menten kinetics [16]). This is the quasi-steady state assumption.

We must compare our choice of k to the inverse characteristic time of change in physiology of our cells. Let us note the latter is not μ, but rather the characteristic time of change of the experimental properties, such as the concentration of species in the culture medium. In an ideal continuous culture, this time should be the whole observation time of the exponential growth, since cell physiology in ideal culture conditions should not change.

The slowest $k = k_{slow}$ we can choose is the ribosome transcription rate for the average peptide. We have:

$$k = k_{slow} = \frac{k_{trans}}{L_{avg}^{aa}} \approx \frac{10}{325} * 3600 \approx 10^2\,\mathrm{h}^{-1}. \quad (8)$$

This yields the characteristic time $t_{slow} = 1/k_{slow} = 10^{-2}\,\mathrm{h} \approx 30\,\mathrm{s}$. As long as the characteristic time of change in physiology is longer than the slowest mode of our system, the steady state assumption holds. Since k_{slow} lowerbounds all the other rate constants of the system, if the steady state assumption is valid for k_{slow}, then the steady-state assumption is valid for all the other parameters and variables of the system. We can then formulate the steady-state assumption:

> As long as the characteristic time of change of the physiology is much longer than 30 s, the steady-state assumption is valid for all variables and parameters of the model.

Dilution rate

We can also use Eq. 7 to study the contribution of each term of the right-hand side of the equation.

In a Michaelis-Menten case, we set $k = k_{cat}$ and rewrite:

$$\left(\frac{1}{k}K\right) \cdot y = \frac{1}{k_{cat}} \cdot \frac{k_{cat}}{c_0} \cdot \Phi(C), \quad (9)$$

$$= \frac{1}{k_{cat} \cdot e_0} \cdot \frac{e_0}{c_0} \cdot k_{cat} \cdot \Phi(C), \quad (10)$$

$$= \frac{e_0}{c_0} \cdot \frac{v}{V_{max}} \quad (11)$$

with K_M, V_{max} the usual Michaelis-Menten constants, v the flux we are studying, and we chose $e_0 = E_{qss}$ the total concentration of the enzyme catalyzing the reaction at quasi-steady state. Since $v/V_{max} \approx 1$ in terms of orders of magnitude, and $z = C_X/c_0 \approx 1$ also, comparing the two right-hand side terms of Eq. 7 is equivalent to comparing e_0/c_0 and μ/k.

In particular, if we consider X to be a metabolite, we can set $k \approx 10^2\,\mathrm{s}^{-1} \approx 10^5\,\mathrm{h}^{-1}$, a typical catalytic rate constant for metabolic reactions, and $c_0 \approx 10^{-2}$ mmol/gDW and $e_0 \approx 10^{-5}$ mmol/gDW according to section , we obtain:

$$\frac{e_0}{c_0} \approx 10^{-3}, \qquad \frac{\mu}{k} \approx 10^{-5}. \tag{12}$$

The dilution term appears to be negligible for metabolites.

In the case of peptides, we must set $k = k_{tsl} \approx 10^2\,\mathrm{h}^{-1}$ (see Eq. 8). The substrates of the translation are amino acids, hence $c_0 \approx 10^{-2}\,\mathrm{mmol_{aa}/gDW}$ and the ribosome concentration is $e_0 \approx 10^{-4}\,\mathrm{mmol/gDW}$ according to section . This yields:

$$\frac{e_0}{c_0} \approx 10^{-2}, \qquad \frac{\mu}{k} \approx 10^{-2}. \tag{13}$$

The dilution term appears to be non-negligible for peptides. A similar reasoning can be performed for mRNA synthesis.

With this argument, we can recover the argument on the orders of magnitude made in section , since comparing e_0/c_0 and μ/k is equivalent to evaluating the following quotient:

$$q = \frac{e_0/c_0}{\mu/k} \approx \frac{V_{max}}{\mu \cdot C_X}, \tag{14}$$

If $q \gg 1$, then the dilution term is negligible in front of the metabolic fluxes. If $q \ll 1$, then the dilution term is preponderent. Finally, if $q \approx 1$, then both terms need to be taken into account. We previously showed that, for metabolites, the dilution term is almost always negligible in front of the metabolic fluxes, a result which we recover in Eq. 12, where $q \gg 1$. We also showed previously that macromolecule dilutions are not always negligible in front of their synthesis rates. We recovered this result for peptides, for which we evaluate $q \approx 1$ in Eq. 13.

Balanced growth hypothesis

The idea of balanced growth was introduced by Monod [17], and has been refined further by Campbell [18]. Monod's approach, based on Hinshelwood's work [19], explains that once past the lag phase, the cells reach a stable enzyme composition:

> "[...] the lag and acceleration phases represent essentially a process of equilibration, the functioning of a regulatory mechanism, by virtue of which a certain enzyme balance inside the cells is attained."
>
> Monod J., *The growth of bacterial cultures*

Campbell refines this definition in his work from 1957 [18] by the following statement:

> "[...] growth is balanced over a time interval if, during that interval, every extensive property of the growing system increases by the same factor."
>
> Campbell A., *Synchronization of cell division*

Using this definition, and accounting for the fact that species concentrations inside the cells are the quotient of the extensive factors mass (of the said species) and volume (of the cell), we obtain directly that intracellular concentrations are constant under the hypothesis of balanced growth. Campbell adds that this approximation is well suited for a continuous culture, and is well approximated in a batch reactor.

ETFL Formulation

Conventions

Supplementary Table 3: Indices used in the formulation.

Index letter	Indexed variables	Indexing set
i	Metabolite	$\mathcal{I}$
aa_i	Amino acid	$\mathcal{A}$
j	Reaction/Flux/Enzyme	$\mathcal{J}$
l	Gene/Peptide/mRNA	$\mathcal{L}$
s	Binary coefficient for growth discretization	$\mathcal{S} = \{0..\lceil \log_2 N \rceil\}$
u	Binary coefficient for interpolation discretization	$\mathcal{U} = \{0..N\}$

Supplementary Table 4: Variables used in the formulation.

Symbol	Variable	Unit
μ	Growth rate	h^{-1}
$v_j^{\pm}$	j^{th} net positive/negative biochemical flux	$\mathrm{mmol.gDW}^{-1}.\mathrm{h}^{-1}$
E_j	Concentration of the jth enzyme	$\mathrm{mmol.gDW}^{-1}$
F_l	Concentration of the lth mRNA	$\mathrm{mmol.gDW}^{-1}$
P_l	Concentration of the RNA polymerase assigned to the lth mRNA	$\mathrm{mmol.gDW}^{-1}$
R_l	Concentration of the ribosome assigned to the lth peptide	$\mathrm{mmol.gDW}^{-1}$
$T^u_{\mathrm{aa}_i}$	Concentration of the ith uncharged tRNA	$\mathrm{mmol.gDW}^{-1}$
$T^c_{\mathrm{aa}_i}$	Concentration of the ith charged tRNA	$\mathrm{mmol.gDW}^{-1}$
v_l^{tsl}	Translation rate of the lth gene	$\mathrm{mmol.gDW}^{-1}.\mathrm{h}^{-1}$
v_l^{tcr}	Transcription rate of the lth gene	$\mathrm{mmol.gDW}^{-1}.\mathrm{h}^{-1}$
v_j^{asm}	Assembly rate of the jth enzyme	$\mathrm{mmol.gDW}^{-1}.\mathrm{h}^{-1}$
v_j^{deg}	Degradation rate of the jth enzyme	$\mathrm{mmol.gDW}^{-1}.\mathrm{h}^{-1}$
v_l^{deg}	Degradation rate of the lth mRNA	$\mathrm{mmol.gDW}^{-1}.\mathrm{h}^{-1}$
$v_{\mathrm{aa}_i}^{\mathrm{charging}}$	Charging rate of the ith tRNA	$\mathrm{mmol.gDW}^{-1}.\mathrm{h}^{-1}$

Supplementary Table 5: Parameters used in the formulation.

Symbol	Parameter	Unit
$k_{\text{cat}}^{j,\pm}$	Forward/backward catalytic rate constant of the jth net biochemical flux	h^{-1}
k_{deg}^{j}	Degradation rate constant of the jth enzyme	h^{-1}
k_{deg}^{l}	Degradation rate constant of the lth mRNA	h^{-1}
η_l^j	Stoichiometry of the lth peptide in the jth enzyme	[∅]
$\eta_{\text{aa}_i}^l$	Stoichiometry of the ith amino acid in the lth peptide	[∅]
L_l^{aa}	Length in amino acids (aa) of the lth peptide	aa
L_l^{nt}	Length in nucleotides (nt) of the lth mRNA	b
$L_{\text{rib}}^{\text{nt}}$	Ribosome footprint size on mRNA, in nucleotides	b
ρ	Ribosome occupancy	[∅]
π	RNA polymerase occupancy	[∅]

Bilinear formulation

$$
\begin{aligned}
\underset{\mu, v, E, F, R, P, T}{\text{maximize}} \quad & \mu \\
\text{subject to} \quad & S \cdot v = 0, \\
& v_j^+ - k_{\text{cat}}^{j,+} E_j \leq 0, \quad \forall j \in \mathcal{J}, \\
& v_j^- - k_{\text{cat}}^{j,-} E_j \leq 0, \quad \forall j \in \mathcal{J}, \\
& v_l^{\text{tsl}} - \sum_{j \in \mathcal{J}} \eta_l^j \cdot v_j^{\text{asm}} = 0, \quad \forall l \in \mathcal{L}, \\
& v_{\text{rRNA}_l}^{\text{tcr}} - v_{\text{rib}}^{\text{asm}} = 0, \quad \forall l \in \mathcal{L}, \\
& v_j^{\text{asm}} - v_j^{\text{deg}} - \mu * E_j = 0, \quad \forall j \in \mathcal{J}, \\
& v_l^{\text{tcr}} - v_l^{\text{deg}} - \mu * F_l = 0, \quad \forall l \in \mathcal{L}, \\
& -v_{\text{aa}_i}^{\text{charging}} + \sum_{l \in \mathcal{L}} \eta_{\text{aa}_i}^l \cdot v_l^{\text{tsl}} - \mu * T_{\text{aa}_i}^u = 0, \quad \forall \text{aa}_i \in \mathcal{A}, \\
& v_{\text{aa}_i}^{\text{charging}} - \sum_{l \in \mathcal{L}} \eta_{\text{aa}_i}^l \cdot v_l^{\text{tsl}} - \mu * T_{\text{aa}_i}^c = 0, \quad \forall \text{aa}_i \in \mathcal{A}, \\
& v_j^{\text{deg}} - k_{\text{deg}}^j \cdot E_j = 0, \quad \forall j \in \mathcal{J}, \\
& v_l^{\text{deg}} - k_{\text{deg}}^l \cdot F_l = 0, \quad \forall l \in \mathcal{L}, \\
& v_l^{\text{tcr}} - \frac{k_{cat}^{\text{RNAP}}}{L_l^{\text{nt}}} P_l \leq 0, \quad \forall l \in \mathcal{L}, \\
& v_l^{\text{tsl}} - \frac{k_{\text{cat}}^{\text{rib}}}{L_l^{\text{aa}}} R_l \leq 0, \quad \forall l \in \mathcal{L}, \\
& R_l - \frac{L_l^{\text{nt}}}{L_{\text{rib}}^{\text{nt}}} F_l \leq 0, \quad \forall l \in \mathcal{L}, \\
& \sum_{l \in \mathcal{L}} R_l + R_{\text{F}} - E_{\text{rib}} = 0, \\
& \sum_{l \in \mathcal{L}} P_l + P_{\text{F}} - E_{\text{RNAP}} = 0 = 0, \\
& R_{\text{F}} - (1 - \rho) E_{\text{rib}} = 0, \\
& P_{\text{F}} - (1 - \pi) E_{\text{RNAP}} = 0.
\end{aligned} \tag{1}
$$

Integer-linearized formulation

$$
\begin{aligned}
\underset{\mu, v, E, F, R, P, T}{\text{maximize}} \quad & \mu \\
\text{subject to} \quad & S \cdot v = 0, \\
& v_j^+ - k_{\text{cat}}^{j,+} E_j \leq 0, \quad \forall j \in \mathcal{J}, \\
& v_j^- - k_{\text{cat}}^{j,-} E_j \leq 0, \quad \forall j \in \mathcal{J}, \\
& v_l^{\text{tsl}} - \sum_{j \in \mathcal{J}} \eta_l^j \cdot v_j^{\text{asm}} = 0, \quad \forall l \in \mathcal{L}, \\
& v_{\text{rRNA}_l}^{\text{tcr}} - v_{\text{rib}}^{\text{asm}} = 0, \quad \forall l \in \mathcal{L}, \\
& v_j^{\text{asm}} - v_j^{\text{deg}} - \mu * E_j = 0, \quad \forall j \in \mathcal{J}, \\
& v_l^{\text{tcr}} - v_l^{\text{deg}} - \mu * F_l = 0, \quad \forall l \in \mathcal{L}, \\
& -v_{\text{aa}_i}^{\text{charging}} + \sum_{l \in \mathcal{L}} \eta_{\text{aa}_i}^l \cdot v_l^{\text{tsl}} - \mu * T_{\text{aa}_i}^u = 0, \quad \forall \text{aa}_i \in \mathcal{A}, \\
& v_{\text{aa}_i}^{\text{charging}} - \sum_{l \in \mathcal{L}} \eta_{\text{aa}_i}^l \cdot v_l^{\text{tsl}} - \mu * T_{\text{aa}_i}^c = 0, \quad \forall \text{aa}_i \in \mathcal{A}, \\
& v_j^{\text{deg}} - k_{\text{deg}}^j \cdot E_j = 0, \quad \forall j \in \mathcal{J}, \\
& v_l^{\text{deg}} - k_{\text{deg}}^l \cdot F_l = 0, \quad \forall l \in \mathcal{L}, \\
& v_l^{\text{tcr}} - \frac{k_{\text{cat}}^{\text{RNAP}}}{L_l^{\text{nt}}} P_l \leq 0, \quad \forall l \in \mathcal{L}, \\
& v_l^{\text{tsl}} - \frac{k_{\text{cat}}^{\text{rib}}}{L_l^{\text{aa}}} R_l \leq 0, \quad \forall l \in \mathcal{L}, \\
& R_l - \frac{L_l^{\text{nt}}}{L_{\text{rib}}^{\text{nt}}} F_l \leq 0, \quad \forall l \in \mathcal{L}, \\
& \sum_{l \in \mathcal{L}} R_l + R_{\text{F}} - E_{\text{rib}} = 0, \\
& \sum_{l \in \mathcal{L}} P_l + P_{\text{F}} - E_{\text{RNAP}} = 0 = 0, \\
& R_{\text{F}} - (1-\rho) E_{\text{rib}} = 0, \\
& P_{\text{F}} - (1-\pi) E_{\text{RNAP}} = 0, \\
& \sum_{j \in \mathcal{J}} \text{MW}_j \cdot E_j - \sum_{u \in \mathcal{U}} \lambda_u \cdot P_u^m = 0, \\
& \sum_{l \in \mathcal{L}} \text{MW}_l \cdot F_l - \sum_{u \in \mathcal{U}} \lambda_u \cdot R_u^m = 0, \\
& \text{MW}_{\text{DNA}} \cdot \text{DNA} - \sum_{u \in \mathcal{U}} \lambda_u \cdot \text{Dm}_u = 0,
\end{aligned} \tag{2}
$$

[see next page]

$$
\begin{aligned}
\underset{\mu, v, E, F, R, P, T}{\text{maximize}} \quad & \mu \\
\text{subject to} \quad & \textit{[contd.]} \quad , \\
& \sum_{s=0}^{\lceil \log_2 N \rceil} 2^s \cdot \delta_s \leq N \, , \\
& \mu - \widehat{\mu} \leq \frac{p}{N}, \\
& \widehat{\mu} - \mu \leq \frac{p}{N}, \\
& \sum_{u \in \mathcal{U}} \lambda_u = 1 \quad , \\
& \sum_{u \in \mathcal{U}} u \cdot \lambda_u - \sum_{l \in \mathcal{L}} 2^l \cdot \delta_l = 0 \quad , \\
& E_j + M \cdot \delta_s - z_j^s \leq M, \quad \forall j \in \mathcal{J}, \\
& z_j^s - M \cdot \delta_s \leq 0 \quad , \quad \forall j \in \mathcal{J}, \\
& z_j^s - E_j \leq 0 \quad , \quad \forall j \in \mathcal{J}, \\
& F_l + M \cdot \delta_s - z_l^s \leq M, \quad \forall l \in \mathcal{L}, \\
& z_l^s - M \cdot \delta_s \leq 0 \quad , \quad \forall l \in \mathcal{L}, \\
& z_l^s - F_l \leq 0 \quad , \quad \forall l \in \mathcal{L}, \\
& T_{\mathrm{aa}_i}^u + M \cdot \delta_s - z_{\mathrm{aa}_i}^{u,s} \leq M, \quad \forall \mathrm{aa}_i \in \mathcal{A}, \\
& z_{\mathrm{aa}_i}^{u,s} - M \cdot \delta_s \leq 0 \quad , \quad \forall \mathrm{aa}_i \in \mathcal{A}, \\
& z_{\mathrm{aa}_i}^{u,s} - T_{\mathrm{aa}_i}^u \leq 0 \quad , \quad \forall \mathrm{aa}_i \in \mathcal{A}, \\
& T_{\mathrm{aa}_i}^c + M \cdot \delta_s - z_{\mathrm{aa}_i}^{c,s} \leq M, \quad \forall \mathrm{aa}_i \in \mathcal{A}, \\
& z_{\mathrm{aa}_i}^{c,s} - M \cdot \delta_s \leq 0 \quad , \quad \forall \mathrm{aa}_i \in \mathcal{A}, \\
& z_{\mathrm{aa}_i}^{c,s} - T_{\mathrm{aa}_i}^c \leq 0 \quad , \quad \forall \mathrm{aa}_i \in \mathcal{A}
\end{aligned} \tag{3}
$$

ETFL Glossary

Big-M value A value that is systematically bigger than the other variables in presence within an expression. Used with binary variables to model `if`-type logical dependencies in an optimization problem. Often annotated M in expressions.

Bilinear(ity) A function is said to be bilinear if it contains a product of two of its variables. This term is called a bilinearity. A problem with a constraint defined by a blinear function of variables is said to be bilinear. That is the case in the non-linearized expression problem with the term $\mu * E_j$, where both μ and E_j are variables of the problem.

Binary variable An integer variable whose value is constrained to 0 or 1. Used to model `if`-type logical dependencies in an optimization problem. For instance, they are used in TFA to enforce the statement "if the Gibbs free energys of this reaction is negative, its net biochemical flux will be in its forward direction". Inclusion of binary variables in a LP problem make it MILP.

Discretization Process by which a continuous variable is replaced by a set of representative discrete values it can take. We use it in ETFL to approximate μ and perform a linearization. Sampling is a type of discretization.

Linearization Process by which a non-linear function is approximated by a linear approximant. In the case of ETFL, we discretize μ to make the bilinear terms $\mu * E_j$ (piecewise-)linear.

LP Linear program. An optimization formulation where a problem is defined by a linear objective function, a set of linear equalities and a set of linear inequalities. FBA is a kind of LP.

MILP A LP with integer variables. The problem is then piecewise-linear, and requires specific solving methods. When all the integer variables are fixed, a LP is obtained. TFA is a kind of MILP.

Special Ordered Set of type 1 (SOS1) constraint A type of constraint where a sum of binary variables has to be lower than or equal to 1. Useful to model a choice between different possibilities.

Zeroth order approximation Approximation of a function using a piecewise constant function. The values of the zeroth-order approximation of the function are a discretization of the space of values of the initial function.

Supplementary References

[1] Salvy P, Fengos G, Ataman M, Pathier T, Soh KC, Hatzimanikatis V. pyTFA and matTFA: A Python package and a Matlab toolbox for Thermodynamics-based Flux Analysis [Journal Article]. Bioinformatics. 2018;.

[2] Dalke A, Wilczynski B, Chapman BA, Cox CJ, Kauff F, Friedberg I, et al. Biopython: freely available Python tools for computational molecular biology and bioinformatics. Bioinformatics. 2009 03;25(11):1422–1423. Available from: `https://dx.doi.org/10.1093/bioinformatics/btp163`.

[3] Caspi R, Foerster H, Fulcher CA, Kaipa P, Krummenacker M, Latendresse M, et al. The MetaCyc Database of metabolic pathways and enzymes and the BioCyc collection of Pathway/Genome Databases. Nucleic acids research. 2007;36(suppl_1):D623–D631.

[4] Keseler IM, Collado-Vides J, Gama-Castro S, Ingraham J, Paley S, Paulsen IT, et al. EcoCyc: a comprehensive database resource for Escherichia coli. Nucleic acids research. 2005;33(suppl_1):D334–D337.

[5] Travers M, Paley SM, Shrager J, Holland TA, Karp PD. Groups: knowledge spreadsheets for symbolic biocomputing. Database. 2013;2013.

[6] Fredrickson A. Formulation of structured growth models. Biotechnology and bioengineering. 1976;18(10):1481–1486.

[7] Bennett BD, Kimball EH, Gao M, Osterhout R, Van Dien SJ, Rabinowitz JD. Absolute metabolite concentrations and implied enzyme active site occupancy in Escherichia coli. Nature chemical biology. 2009;5(8):593.

[8] Bartholomäus A, Fedyunin I, Feist P, Sin C, Zhang G, Valleriani A, et al. Bacteria differently regulate mRNA abundance to specifically respond to various stresses. Philosophical Transactions of the Royal Society A: Mathematical, Physical and Engineering Sciences. 2016;374(2063):20150069.

[9] Taniguchi Y, Choi PJ, Li GW, Chen H, Babu M, Hearn J, et al. Quantifying E. coli proteome and transcriptome with single-molecule sensitivity in single cells. Science. 2010;329(5991):533–538.

[10] Piques M, Schulze WX, Höhne M, Usadel B, Gibon Y, Rohwer J, et al. Ribosome and transcript copy numbers, polysome occupancy and enzyme dynamics in Arabidopsis. Molecular systems biology. 2009;5(1).

[11] Bremer H, Dennis PP. Modulation of chemical composition and other parameters of the cell by growth rate [Journal Article]. Escherichia coli and Salmonella: cellular and molecular biology. 1996;2(2):1553–69.

[12] Dill KA, Ghosh K, Schmit JD. Physical limits of cells and proteomes. Proceedings of the National Academy of Sciences. 2011;108(44):17876–17882.

[13] Kaleta C, Schäuble S, Rinas U, Schuster S. Metabolic costs of amino acid and protein production in Escherichia coli. Biotechnology journal. 2013;8(9):1105–1114.

[14] Stephanopoulos G, Aristidou AA, Nielsen J. Metabolic engineering: principles and methodologies. Elsevier; 1998.

[15] Benyamini T, Folger O, Ruppin E, Shlomi T. Flux balance analysis accounting for metabolite dilution. Genome biology. 2010;11(4):R43.

[16] Heineken F, Tsuchiya H, Aris R. On the mathematical status of the pseudo-steady state hypothesis of biochemical kinetics. Mathematical Biosciences. 1967;1(1):95–113.

[17] Monod J. The growth of bacterial cultures. Annual review of microbiology. 1949;3(1):371–394.

[18] Campbell A. Synchronization of cell division. Bacteriological reviews. 1957;21(4):263.

[19] Hinshelwood CN. The chemical kinetics of the bacterial cell.; 1946.
