# Supplementary material for: The ETFL formulation allows multi-omics integration in thermodynamics-compliant metabolism and expression models
Source: Nat Commun. 2020 Jan 13;11:30. doi: 10.1038/s41467-019-13818-7 (PMC6959363; doi:10.1038/s41467-019-13818-7)
Supplement: Supplementary file 2 — Description of Additional Supplementary Files [file 41467_2019_13818_MOESM2_ESM.pdf]

Description of Additional Supplementary Files

File Name: Supplementary Data 1

Description: Complement on the gene essentiality study. List of mismatches in the essentiality of genes between iJO1366 predictions and vETFL predictions. Count summarized per subsystem, and transition matrix of iJO1366 prediction to ETFL prediction for the mismatches.
