# Supplementary material for: The ETFL formulation allows multi-omics integration in thermodynamics-compliant metabolism and expression models
Source: Nat Commun. 2020 Jan 13;11:30. doi: 10.1038/s41467-019-13818-7 (PMC6959363; doi:10.1038/s41467-019-13818-7)
Supplement: Supplementary file 4 — Reporting Summary [file 41467_2019_13818_MOESM4_ESM.pdf]

nature research

Corresponding author(s): Vassily Hatzimanikatis

Last updated by author(s): Nov 14, 2019

Reporting Summary

Nature Research wishes to improve the reproducibility of the work that we publish. This form provides structure for consistency and transparency in reporting. For further information on Nature Research policies, see Authors & Referees and the Editorial Policy Checklist.

Statistics

For all statistical analyses, confirm that the following items are present in the figure legend, table legend, main text, or Methods section.

n/a	Confirmed	
☐	☒	The exact sample size (*n*) for each experimental group/condition, given as a discrete number and unit of measurement
☐	☒	A statement on whether measurements were taken from distinct samples or whether the same sample was measured repeatedly
☒	☐	The statistical test(s) used AND whether they are one- or two-sided<br>*Only common tests should be described solely by name; describe more complex techniques in the Methods section.*
☒	☐	A description of all covariates tested
☐	☒	A description of any assumptions or corrections, such as tests of normality and adjustment for multiple comparisons
☐	☒	A full description of the statistical parameters including central tendency (e.g. means) or other basic estimates (e.g. regression coefficient) AND variation (e.g. standard deviation) or associated estimates of uncertainty (e.g. confidence intervals)
☒	☐	For null hypothesis testing, the test statistic (e.g. *F*, *t*, *r*) with confidence intervals, effect sizes, degrees of freedom and *P* value noted<br>*Give P values as exact values whenever suitable.*
☒	☐	For Bayesian analysis, information on the choice of priors and Markov chain Monte Carlo settings
☒	☐	For hierarchical and complex designs, identification of the appropriate level for tests and full reporting of outcomes
☒	☐	Estimates of effect sizes (e.g. Cohen's *d*, Pearson's *r*), indicating how they were calculated

Our web collection on statistics for biologists contains articles on many of the points above.

Software and code

Policy information about availability of computer code

Data collection	Custom code has been written in Python and is available on our GitHub/GitLab repository. Other data has been obtained from publications, for which the references are provided in the main text and a copy has been included in the organism_data/ folder of our repository.
Data analysis	Means (geometric and arithmetic), medians, have been calculated using the Python packages numpy and scikit-learn.<br>The repositories contain all the code to reproduce all the results presented in the paper, including the models.

For manuscripts utilizing custom algorithms or software that are central to the research but not yet described in published literature, software must be made available to editors/reviewers. We strongly encourage code deposition in a community repository (e.g. GitHub). See the Nature Research guidelines for submitting code & software for further information.

Data

Policy information about availability of data

All manuscripts must include a data availability statement. This statement should provide the following information, where applicable:

- Accession codes, unique identifiers, or web links for publicly available datasets
- A list of figures that have associated raw data
- A description of any restrictions on data availability

All the data used for generating the figures can be reproduced with the scripts provided in the repository. All data taken from other publications is documented in the main text, and the code comments also includes links and identifiers to these sources.

Field-specific reporting

Please select the one below that is the best fit for your research. If you are not sure, read the appropriate sections before making your selection.

☒ Life sciences ☐ Behavioural & social sciences ☐ Ecological, evolutionary & environmental sciences

For a reference copy of the document with all sections, see nature.com/documents/nr-reporting-summary-flat.pdf

Life sciences study design

All studies must disclose on these points even when the disclosure is negative.

Sample size	No samples other than simulation times were taken. Our simulation times are reported against claimed approximate published values. For this we thought sample sizes from 190 to 8000 were sufficient.
Data exclusions	We report only the results of the most recent calculations made with the most recent code for the preparation of the manuscript. Some simulations were repeated with different solver parameters in case of solver difficulties (eg. timeout), in which case we report only successful optimizations.
Replication	MILP optimal values are a global optimum, and are thus mathematically guaranteed to be replicated. Some simulations were repeated with different solver parameters in case of solver difficulties (eg. timeout), until optimality was reached. Solution variability at optimality is discussed and addressed using variation analysis.
Randomization	There is no step in the formulation where randomization can be used.
Blinding	There is no step in the formulation where blinding can be used.

Reporting for specific materials, systems and methods

We require information from authors about some types of materials, experimental systems and methods used in many studies. Here, indicate whether each material, system or method listed is relevant to your study. If you are not sure if a list item applies to your research, read the appropriate section before selecting a response.

Materials & experimental systems

n/a	Involved in the study	
☒	☐	Antibodies
☒	☐	Eukaryotic cell lines
☒	☐	Palaeontology
☒	☐	Animals and other organisms
☒	☐	Human research participants
☒	☐	Clinical data

Methods

n/a	Involved in the study	
☒	☐	ChIP-seq
☒	☐	Flow cytometry
☒	☐	MRI-based neuroimaging
